# Dispersal Across Headwaters Determines Fish Population Structure Between Interdigitating River Systems in the Guiana Shield Highlands

**DOI:** 10.1002/ece3.73603

**Published:** 2026-05-21

**Authors:** Thomas D. Morgan, Karen M. Alofs, Donald C. Taphorn, Devya Hemraj‐Naraine, Elford Liverpool, Jan Mol, Raphael Covain, Nathan K. Lujan, Hernán López‐Fernández

**Affiliations:** ^1^ Ecology and Evolutionary Biology Department University of Michigan Ann Arbor Michigan USA; ^2^ Forensic Science Trent University Peterborough Ontario Canada; ^3^ School for Environment and Sustainability University of Michigan Ann Arbor Michigan USA; ^4^ Center for the Study of Biological Diversity University of Guyana Georgetown Guyana; ^5^ Department of Biology University of Louisville Louisville Kentucky USA; ^6^ Department of Biology University of Guyana Georgetown Guyana; ^7^ Department of Biology Anton de Kom Universiteit van Suriname Paramaribo Suriname; ^8^ Muséum d'Histoire Naturelle, Genève Vertébrés – Ichtyologie Genève Switzerland; ^9^ Royal Ontario Museum Toronto Ontario Canada; ^10^ Ecology and Evolutionary Biology University of Toronto Toronto Ontario Canada; ^11^ Museum of Zoology University of Michigan Ann Arbor Michigan USA

## Abstract

Riverine aquatic species primarily occupy the dendritic networks that define river basins, and population‐structure models for riverine taxa, including freshwater fishes, generally assume strictly longitudinal dispersal within these networks. River configurations, however, are dynamic: hydrologic connections change seasonally and over geologic time, potentially enabling *out‐of‐network* dispersal, a process that remains poorly studied. Because such dispersal violates key assumptions of existing models, its prevalence may require the development of new frameworks for understanding riverine population structure. To test for out‐of‐network connectivity between interdigitating river systems, we analyzed genetic population structure in *Krobia potaroensis*, a cichlid fish endemic to the rivers draining the Pakaraima Mountains in western Guyana, South America. We detected genetic associations among populations that do not correspond to contemporary river configurations, indicating historical or ongoing dispersal between adjacent river systems. These results demonstrate that within‐network longitudinal dispersal cannot always be assumed and that alternative dispersal routes can shape population structure, genetic diversity, and genetic differentiation in riverine taxa.

## Graphical abstract

The genetic population structure of riverine fishes is strongly shaped by the architecture of dendritic river networks, which can shift over both contemporary and geological timescales. Such river rearrangements are rarely incorporated into models of riverscape genetics, but they can have a profound effect on fish populations, especially when formerly distinct drainage systems connect and permit inter‐network dispersal. We investigated the genetic structure of the Neotropical cichlid *Krobia potaroensis* and found evidence of inter‐network dispersal, underscoring the importance of considering these dynamic hydrological connections.

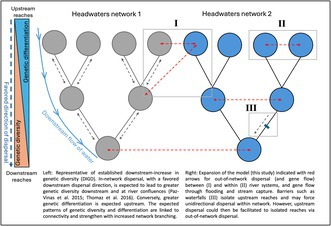

## 1 Introduction

Population connectivity and genetic diversity critically influence the ecology, evolution, and persistence of species (Gilpin and Soulé 1986; Soulé 1987). Genetic diversity and how it is distributed within a heterogeneous spatial environment affects the fitness and resilience of populations, and has long been incorporated into biogeographic studies and conservation planning strategies (Allendorf et al. 2010; DeWoody et al. 2021; Gilpin and Soulé 1986). Moreover, the distribution and evolutionary trajectories of species can strongly depend on their ability to disperse across the landscape (Kodandaramaiah 2009), whether between subpopulations of the established species distribution, or by moving into novel systems with subsequent range expansion. Conversely, population fragmentation can lead to disconnected and smaller subpopulations, which are in turn more likely to differentiate in allopatry or become locally extinct causing either extirpation or range contraction (Lanfear et al. 2014; Shaffer and Samson 1985). Therefore, connectivity, both contemporarily and over geologic or evolutionary time, fundamentally affects the evolution and persistence of natural populations.

For riverine taxa such as fishes, river network architecture structures the connectivity of populations on contemporary timescales (Blanchet et al. 2020; Borthagaray et al. 2025; Fagan 2002; Labonne et al. 2008; Meffe and Vrijenhoek 1988; Paz‐Vinas et al. 2015; Thomaz et al. 2016; Vannote et al. 1980), and shapes species distributions on macroevolutionary timescales (Albert et al. 2011; Gilbert 1980; Lundberg et al. 1998; Ribas et al. 2025). Several models of rivers as dendritic networks have been developed to describe the varying connectivity and structure of metapopulations from small upland tributaries downstream to large downstream channels (Altermatt et al. 2013; Fagan 2002; Labonne et al. 2008; Lujan and Conway 2015; Paz‐Vinas and Blanchet 2015; Thomaz et al. 2016; Tonkin et al. 2018). Disruptions to inter and intra‐network connectivity, can reduce aquatic dispersal and gene flow, and shape regional patterns of freshwater biogeography (Gilbert 1980). Extensions on the dendritic model have shown that both resource and habitat availability change based on riverine architecture, and that biodiversity and species abundance are consequently modified along a river's branches and at their confluences (Campbell Grant et al. 2007; Paz‐Vinas and Blanchet 2015; Shao et al. 2019). Increased habitat availability and habitat heterogeneity found in the downstream branches of river networks and at their nodes result in greater downstream genetic and species diversity (α‐diversity) relative to upstream reaches (Paz‐Vinas et al. 2015; Thomaz et al. 2016; Vannote et al. 1980). Accordingly, more isolated upstream systems are hypothesized to have lower genetic and α‐diversity, and higher genetic differentiation than downstream reaches. This model has been described as the ‘downstream increase in genetic diversity’ (DIGD) expectation within rivers (Box 1) (Paz‐Vinas et al. 2015; Thomaz et al. 2016). The varied structure of fish communities and populations along river systems reflects the differing but critical influence of connectivity on riverine biodiversity. Greater biomass and overall biodiversity are predicted and observed within the larger downstream reaches of river basins (Fisher 1997; Matthews 1986; Vannote et al. 1980). However, upstream and headwater systems constitute most of the length of river systems (typically > 75% of total river length), and often contain upstream‐endemic species, thus contributing substantially to riverine β‐ and Γ‐diversity (Finn et al. 2011).

BOX 1 **Conceptual model of river network connectivity and gene flow.**

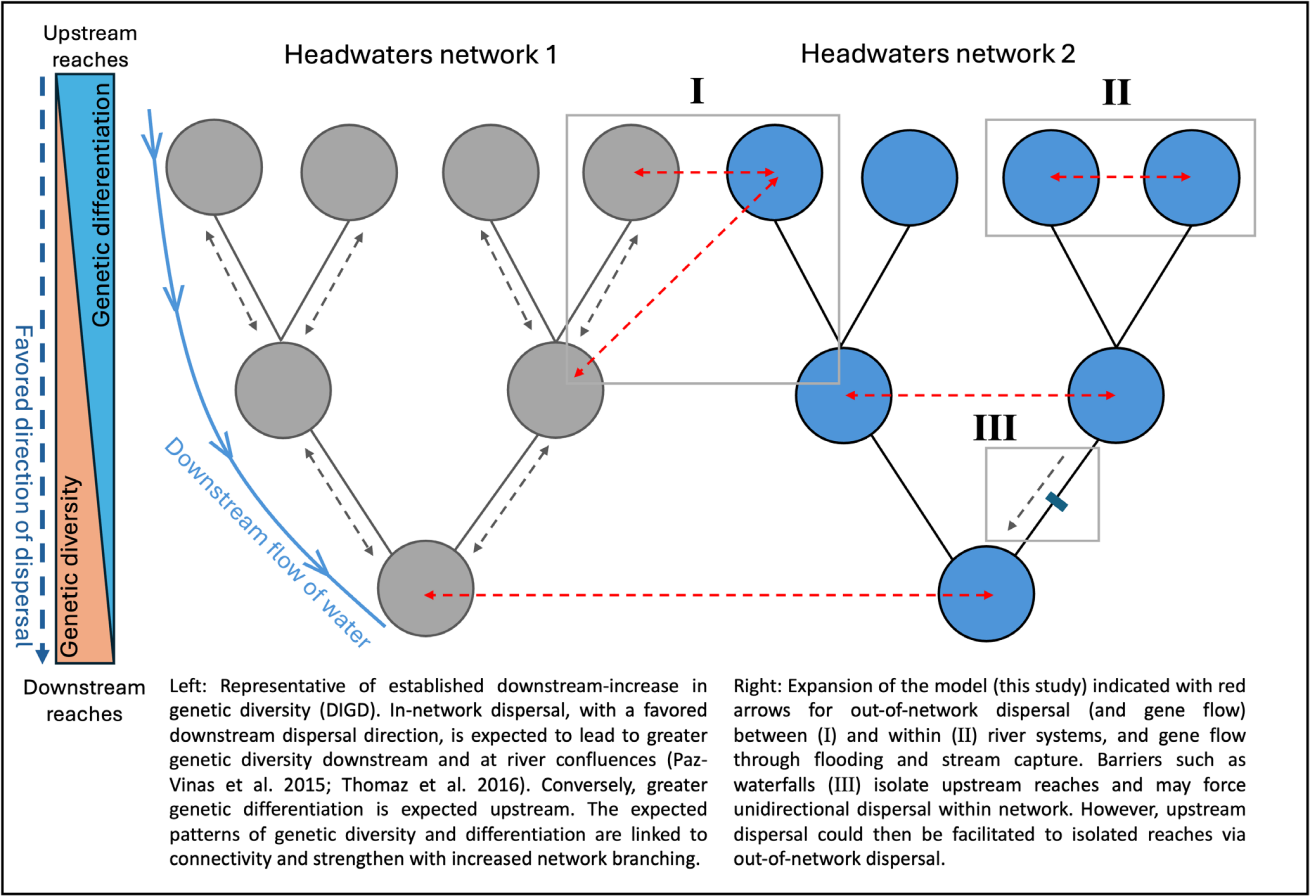

Existing models of riverine population structure typically consider single dendritic networks and restrict the dispersal of aquatic organisms (i.e., fishes, aquatic invertebrates, and other taxa) to the wetted channel (‘within‐network’; see Box 1). However, out‐of‐network hydrologic connections can occur both within a wider system, such as when flooding causes a river and its tributaries (the ‘river system’) to temporarily lose their network architecture (‘intra‐system’ + ‘out‐of‐network’ connections), or between otherwise distinct river systems or basins (‘inter‐system’ + ‘out‐of‐network’ connections). These hydrologic connections can allow fishes to disperse outside the defined river network, connecting otherwise distinct populations both contemporarily (De Souza et al. 2012, 2020; Stoffels et al. 2016; Willis et al. 2010) and over geologic time (Burridge et al. 2006; Lujan and Armbruster 2011; Thomaz and Knowles 2020; Waters et al. 2020, 2026). Understanding when and how aquatic organisms use inter‐system conduits is essential for expanding riverine population models to account for out‐of‐network and inter‐system dispersal.

Accounting for out‐of‐network and inter‐system connectivity is particularly important because river basins are not immutable in their architecture and can naturally change their configurations over ecological (e.g., transient flooding) and geological time (e.g., river avulsions or river capture). Such processes redefine the catchments of river basins and the configuration of upland tributaries, ultimately changing the downstream systems to which they flow (Bishop 1995; Slingerland and Smith 2004). River systems may also change their flow seasonally (e.g., due to flooding/inundation), augmenting the dispersal environment within and between river systems (e.g., rapids and waterfalls or floodplains being inundated, facilitating seasonal dispersal; see Box 1). Out‐of‐network dispersal routes may have a critical effect on the connectivity and resilience of populations by modulating the effects of barriers (e.g., rapids or waterfalls), as well as altering the fundamental assumption that upstream riverine demes are the most isolated in riverine metapopulations.

Analyzing instances where inter‐system (and therefore out‐of‐network and ‘overland’) dispersal of fishes may have occurred requires sampling adjacent river systems at a regional scale, encompassing the broader distribution of focal populations. However, empirical studies of genetic population structure and genetic diversity in rivers have principally focused on smaller geographic scales, such as subsets of river systems (Shao et al. 2019); which can limit or preclude observation of inter‐system dispersal. Studies of species that are distributed across adjacent and interdigitating river systems should uniquely illustrate how out‐of‐network dispersal conduits affect population distribution and structure within and between adjacent river networks. Differentiating between instances where out‐of‐network dispersal has occurred from instances better described by models of within‐network movement should greatly clarify the evolutionary and biogeographic history of riverine species. Moreover, improved dispersal models for riverine taxa should strengthen conservation and management through improved delineation of population boundaries and their stability (Campbell Grant et al. 2010; Csermely 2004).

**FIGURE 1:**
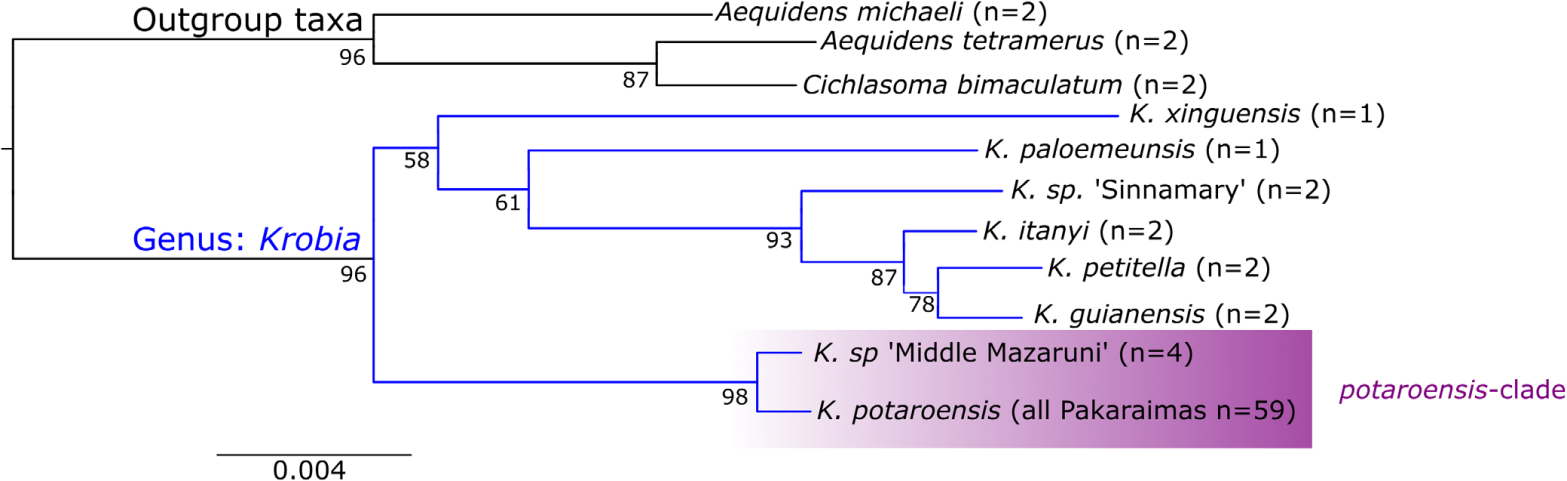
Phylogeny of *Krobia* (blue) and selected outgroups (black). Tree generated from RAxML analysis of 2,107,645 bp representing 7440 loci with 1000 bootstrap replicates. The analyzed ‘potarensis‐clade’ *Krobia* (purple) is shown relative to congeners and closely related outgroups.

**FIGURE 2:**
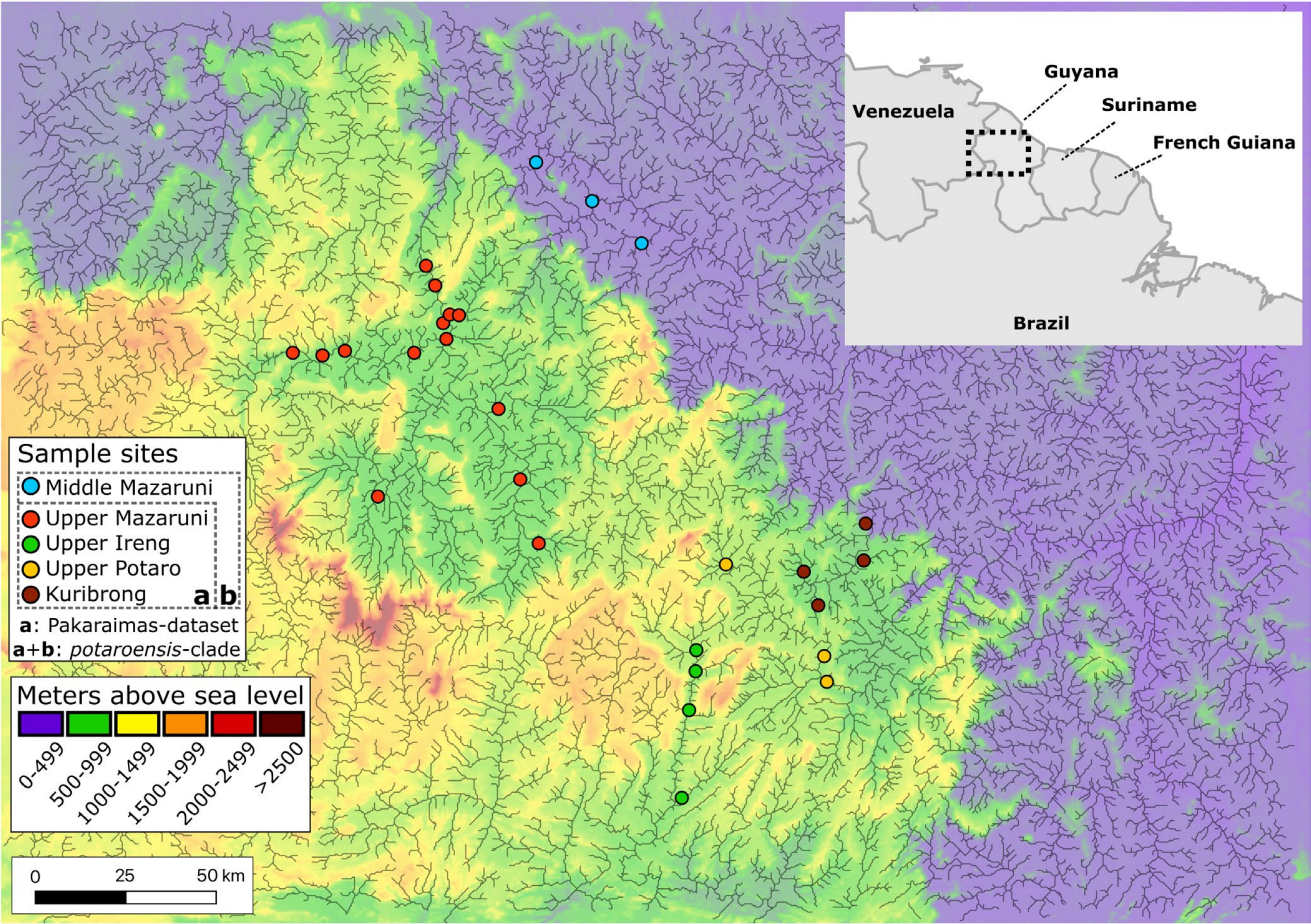
Sampling sites for *Krobia potaroensis* (*n* = 59) from the river systems of the Pakaraima Mountains and sister *Krobia* sp. ‘Middle‐Mazaruni’ (*n* = 4). Map generated in QGIS v3 with topography layers derived from NASA Shuttle Radar Topography Mission Global 1 arc second (2013), and river layers from the free‐flowing rivers dataset (Grill et al. 2019). Due to mapping scale, one symbol may represent two or more nearby localities.

Fish dispersal, as facilitated by both inter‐ and intra‐system hydrologic connectivity, can be better understood by analyzing the population structure of species that exist among multiple adjacent river networks. The Pakaraima Mountains Region in Guyana (Figure 1) is an ideal area where many of the riverine architecture features affecting population structure can be observed. The main rivers draining the Pakaraimas (the upper Mazaruni, upper Ireng, upper Kuribrong and upper Potaro Rivers; Figure 2), are all separated from lower portions of their containing river basins by large rapids or waterfalls, and consequently hold distinct upland fish assemblages (Alofs et al. 2014; Hardman et al. 2002; Lujan et al. 2013; Taphorn et al. 2017). Each of the four river systems of the Pakaraimas harbors high proportions of endemic fishes, with species either isolated within individual river systems (e.g., 65%–95% endemic fish species within the upper Mazaruni (Alofs et al. 2014)), or species that, as currently understood, are distributed throughout the upper tributaries of the Pakaraimas (e.g., *Krobia [= Aequidens] potaroensis* (Eigenmann 1912) or *Trichomycterus cf. guianensis* (Hayes et al. 2020)). Even where species are present within (but not endemic to) the Pakaraima river systems (e.g., *Gymnotus carapo* ), the large waterfalls that separate the upper tributaries from their lower reaches still represent a substantial delineating barrier from the lowland fish communities (Hardman et al. 2002; Lehmberg et al. 2018).

The Neotropical cichlid *Krobia potaroensis* (Subfamily: Cichlinae) is a species putatively found throughout the river systems of the Pakaraimas. Originally described as *Aequidens potaroensis* (Eigenmann 1912), more recent descriptive studies have noted that *‘A’ potaroensis* (along with *‘A’ paloemeuensis*) is distinct in morphologic features from true *Aequidens* and more similar to the genus *Krobia* (Kullander and Nijssen 1989). Molecular phylogenies have unequivocally placed *‘A’ potaroensis* within a clade that includes lowland *Krobia* (and is the sister genus to true *Aequidens* (Ilves et al. 2018; López‐Fernández et al. 2010; Musilová et al. 2009)). We follow those authors and refer to the species as *Krobia potaroensis* throughout this paper. The presence of putative *K. potaroensis* in all four river systems of the Pakaraimas contrasts with the pattern of many species in the region that typically occur only within a subset of the river systems (e.g., *Trichomycterus* sp. (Hayes et al. 2020); *Mazarunia* spp. (López‐Fernández et al. 2012); *Akawaio penak* (Maldonado‐Ocampo et al. 2014); *Yaluwak primus* (Lujan et al. 2019)). *Krobia potaroensis* therefore provides a useful model for understanding whether (and how) the interdigitation of the Pakaraimas river systems (with tributaries < 5 km from one another (Grill et al. 2019)) may contribute to dispersal between these otherwise isolated systems.

We used a ddRAD approach (Peterson et al. 2012) consisting of thousands of genetic loci from individuals within each of the four river systems to analyze the population structure and connectivity of *K. potaroensis* across the interdigitating river systems of the Pakaraimas. We interrogated the dataset to ask: (i) Do putative *K. potaroensis* from throughout the Pakaraimas belong to a single clade; and (ii) what is the genetic population structure within and between the river systems? Answering these questions by characterizing the degree of divergence or of ongoing gene flow, between populations of *K. potaroensis* tests the ability of contemporary models of riverine populations to accurately describe the genetic structure of fish populations within and among river networks and helps identify the processes that lead to multi‐basin distributions of freshwater fishes on timescales relevant to speciation.

Considering the high degree of inter‐system interdigitation, the presence of putative *Krobia potaroensis* within multiple distinct and adjacent river systems, and that both river capture and ephemeral flooding have been well characterized in the wider region, we discuss the observed population structure of *K. potaroensis* in the context of an expanded conceptual model of riverine population connectivity (Box 1) that considers out‐of‐network dispersal.

## 2 Methods

### 2.1 Sampling

A combined total of 79 specimens of *K. potaroensis* (*n* = 63) and selected outgroups (*n* = 16) were obtained from museum collections (Figure 1, Tables 1 and S1). Included samples spanned as much of the upper Mazaruni, Ireng, Kuribrong, and Potaro River basins as possible with the goal of characterizing putative intraspecific variation within and between river systems. Outgroup sampling (*n* = 16) aimed at testing the monophyly of *K. potaroensis* along its native distribution and to explore its relationships to other described *Krobia* species and sister genus *Aequidens*.

**TABLE 1:** Sample details for phylogenetic and population genetic analyses.

Species	Country–Region	River system	Dataset	*N*
*Aequidens michaeli*	Brazil	Xingu River	Phy	2
*Aequidens tetramerus*	Brazil	Rio Novo	Phy	2
*Cichlasoma bimaculatum*	Guyana	Demerara River	Phy	2
*Krobia xinguensis*	Brazil	Xingu River	Phy	1
*Krobia paloemeunsis*	Suriname	Paloemeu River	Phy	1
*Krobia* sp. ‘Sinnamary’	French Guiana	Sinnamary River	Phy	2
*Krobia itanyi*	Suriname	Marowijne‐Maroni River	Phy	2
*Krobia petitella*	Guyana	Berbice River	Phy	2
*Krobia guianensis*	Suriname	Suriname River	Phy	2
*Krobia* sp. ‘Middle Mazaruni’	Guyana	Middle Mazaruni River	Phy, Pot	4
*Krobia potaroensis*	Guyana	Upper Mazaruni, Upper Potaro, Upper Ireng and Kuribrong Rivers	Phy, Pot, Pak	59

*Note:* Full details for each individual sample including museum accession numbers and GPS included in Table S1. Samples were variously included in either the ‘Phy’ = phylogeny dataset, the ‘Pot’ = *potaroensis*‐clade dataset (*n* = 63), and/or the ‘Pak’ = Pakaraimas dataset, as described in text.

### 2.2 ddRAD Sequencing, Demultiplexing, and Matrix Assembly

Reduced representation libraries were sequenced for all individuals using a ddRAD approach (Peterson et al. 2012) modified to use infrequent cut sites and a wide size‐selection window (Sabaj et al. 2020). DNA was first extracted using Qiagen DNeasy Blood and Tissue kits, with the addition of 35 U of RNaseA (Qiagen) and quantified using a Qubit fluorometer. 500 ng of DNA was then digested for each sample using 40U of SphI‐HF and 40U of EcoRI‐HF (New England Biolabs). Following digestion, individual barcodes were introduced through adaptor ligation and individuals pooled into libraries of 40–48 individuals. Pooled libraries were each then size selected for fragments between 375 and 525 bp on a PippenPrep gel electrophoresis system (Sage Science). Illumina adaptors were added using PCR (as in Peterson et al. 2012) and libraries were sequenced using the PE150 chemistry on a NovaSeq platform (Illumina) at the University of Michigan Advanced Genomics Core.

Following sequencing, raw sequence files were demultiplexed and matrices were assembled using *ipyrad* (version 0.9.63, Eaton and Overcast 2020). Loci were exported from *ipyrad* under a de novo alignment, using the default parameters in all cases, except the clustering threshold and the minimum depth threshold, which were set to 0.85 and 6, respectively. Matrices were exported for three datasets including the following samples: (i) Pakaraimas, middle Mazaruni, and outgroups (*n* = 79; hereafter the ‘phylogeny‐dataset’), (ii) Pakaraimas + middle‐Mazaruni (as the closest outgroup to the Pakaraimas; *n* = 63; hereafter the ‘*potaroensis*‐clade’ dataset), and (iii) the upper Pakaraimas (*n* = 59; hereafter the ‘Pakaraimas’ dataset). Matrices were exported for loci present in > 50% of individuals for the phylogeny dataset (to retain more loci in outgroup samples for tree building (Huang and Knowles 2016; Wagner et al. 2013)) and in > 75% of individuals for the *potaroensis*‐clade and Pakaraimas datasets; thus representing loci in at least 40, 48, and 45 individuals for the phylogeny dataset (of the *n* = 79, *n* = 63, and *n* = 59 individuals in the phylogeny, *potaroensis*‐clade, and Pakaraimas‐dataset, respectively). To investigate the influence of missing data thresholds in the *potaroensis*‐clade we also exported unlinked SNP matrices for less strict thresholds (loci in ≥ 15 and ≥ 30 individuals) and conducted parallel analyses of population substructure. Aligned sequence matrices and SNP‐matrices were exported for all three datasets, while unlinked SNP matrices (one SNP per locus) were additionally exported for the *potaroensis*‐clade and the Pakaraimas datasets.

### 2.3 Taxonomy, Genetic Diversity, and Population Structure Analyses of *Krobia potaroensis*

#### 2.3.1 Phylogeny of *Krobia* in the Pakaraimas

To determine whether all putative *K. potaroensis* form a clade within the Pakaraimas, we first constructed a maximum‐likelihood (ML) tree for the phylogeny‐dataset using RAxML (v 8.0, Stamatakis 2014). The ML tree was calculated under a GTR+Γ model, with 20 independent maximum‐likelihood searches, and with 1000 non‐parametric bootstrap replicates to assess topological robustness.

#### 2.3.2 Clustering Analyses and Species Tree Analyses

To determine whether the genetic diversity of *K. potaroensis* is split into identifiable genetic subpopulations, and to relate these subpopulations to modern river systems, we performed cluster analyses on the unlinked SNP matrices for the *potaroensis*‐clade‐dataset and Pakaraimas‐dataset. First, we determined the most likely number of genetic clusters hierarchically using the program *STRUCTURE* (Pritchard et al. 2000); parallelized using *Strauto* (Chhatre and Emerson 2017). The number of clusters (*K*) was determined through comparison of 10 independent runs of *K* = 1–5 with 50,000 (burn‐in) followed by an additional 500,000 MCMC iterations. Convergence between runs was evaluated using *Structure harvester* (Earl and vonHoldt 2012) and the most likely value of *K* was determined using the Evanno method, which evaluates *K* based on the second order rate of change of the likelihood function (Evanno et al. 2005). Independent *STRUCTURE* runs were combined and visualized using *CLUMPAK* (Jakobsson and Rosenberg 2007; Kopelman et al. 2015). Upon determination of the most likely *K*, each subpopulation was run through *STRUCTURE* again to determine additional substructure, until *K* = 1 was determined most likely (Meirmans 2015). To investigate any effect of missing data, we also ran *STRUCTURE* analyses on our alternative SNP‐matrices, exported with differing missing data thresholds (see Figures S3–S5).

To further examine the genetic relationships between river systems and to account for potential incomplete‐lineage sorting and gene‐tree to species‐tree discordance we estimated population trees for full sequences of the *potaroensis*‐clade and Pakaraimas‐dataset. Population trees were estimated under the multispecies coalescent model using *SVDQUARTETS* (Chifman and Kubatko 2014) as implemented in PAUP* (v 4.0) (Swofford 2002). Trees were estimated under all possible quartets, with 1000 bootstrap replicates to assess branch support.

#### 2.3.3 Principal Components Analysis and Measures of Genetic Diversity

We ran a principal components analysis (PCA) and spatial principal components analysis (sPCA) in *Adegenet* v2.1.10 (Jombart and Ahmed 2011) to visualize the genetic diversity within and between river systems, imputing mean values for missing data. To again infer any effect of missing data, we ran our PCA analyses on the three alternative SNP matrices for the Pakaraimas dataset as well as the *potaroensis*‐clade dataset. To represent the genetic diversity within Pakaraimas rivers we then calculated observed heterozygosity (*H*_O_), and within‐population gene diversity (*H*_S_) for the populations of *K. potaroensis* using the R package *hierfstat* v0.5‐11 (Goudet 2005). Measures of population subdivision were calculated in *scikit‐allel* v1.3.5 (Miles et al. 2023) using Hudson's *F*_ST_ method (Hudson et al. 1992), which better represents genetic differentiation when using large SNP datasets (Bhatia et al. 2013). We again used the Pakaraimas dataset (*n* = 59), first imputing any missing data in *Beagle* v4.1 (Browning and Browning 2007), and filtering the data to only include biallelic SNPs using *bcftools* v1.12 (Danecek et al. 2021).

#### 2.3.4 Demographic History Between River Systems

In order to observe how genetic differentiation between river systems corresponds to hypothesized inter‐system connections, we modeled the demographic histories between each population pair in *dadi‐cli* (Gutenkunst et al. 2009; Huang et al. 2021). First, we used easySFS (https://github.com/isaacovercast/easySFS) to convert the *ipyrad* SNP‐matrices to site‐frequency spectra (SFS) and to project the allele frequencies that retain the optimal number of segregating sites (Gutenkunst et al. 2009). We generated the SFS for one SNP per‐locus for the *potaroensis*‐clade dataset, running the pairwise comparisons for five populations (four upland and one middle‐Mazaruni). We analyzed the pairs of populations using six two‐dimensional demographic models from the Portik et al. (2017) model library: strict isolation (no_mig), isolation with population size change (no_mig_size), isolation with symmetric migration (split_mig), isolation with migration (IM), secondary contact with symmetric migration (sec_contact_sym_mig), and secondary contact with asymmetric migration (sec_contact_asym_mig). Model fitting used maximum‐likelihood optimization with parameter bounds of 0.001 to 10 for population size and divergence times, 0–10 for migration rates, 0.01 to 0.99 for ancestral size proportion. As recommended by Portik et al. (2017), we ran three sequential rounds of 50 optimizations each. We seeded each optimization run with the best parameter estimates from the previous round until runs converged. The best performing model for each pairwise comparison was determined by AIC, and parameter uncertainty was estimated using the StatDM function with logscale transformation. We converted the scaled demographic parameters for the best performing model to biological units using a cichlid‐specific mutation rate of 3.5 × 10^−9^ per base per generation (Malinsky et al. 2018) and assuming a generation time of 1 year.

## 3 Results

### 3.1 ddRAD Sequencing, Demultiplexing, and Matrix‐Assembly

The average number of sequenced raw reads per sample was 4,593,480 bp (SD 1,649,438) for samples included in analyses (see Table S2 for individual counts). The ‘phylogeny dataset’ had a final alignment of 2,107,645 bp representing 7440 loci with 18.5% missing data (see Table S2). The sequence‐matrices used in SVDQuartets for the *potaroensis*‐clade‐dataset and the Pakaraimas‐dataset had final alignments of 2,975,799 bp and 3,073,420 bp, respectively, representing 10,366 and 10,719 loci, with 5.2% and 5.0% missing data, respectively. The Pakaraimas dataset SNP matrix initially contained 11,296 SNPs with 8.0% missing sites, which were then filtered to 11,173 biallelic SNPs before calculating Hudson's *F*_ST_. The unlinked SNP matrices exported for the *potaroensis*‐clade‐dataset and the Pakaraimas‐dataset were represented by 7938 and 5100 SNPs, respectively; and with 5.9% and 6.4% missing genotypes, respectively (Table S2). The alternative datasets with differing thresholds of missing data yielded matrices of 6515 and 5895 unlinked SNPS for the Pakaraimas‐dataset for loci in ≥ 15 and ≥ 30 individuals, with missingness of 15.8% and 10.8%, respectively.

### 3.2 Taxonomy, Genetic Diversity, and Population Structure Analyses of *Krobia potaroensis*

#### 3.2.1 Phylogeny of Krobia in the Pakaraimas

**FIGURE 3:**
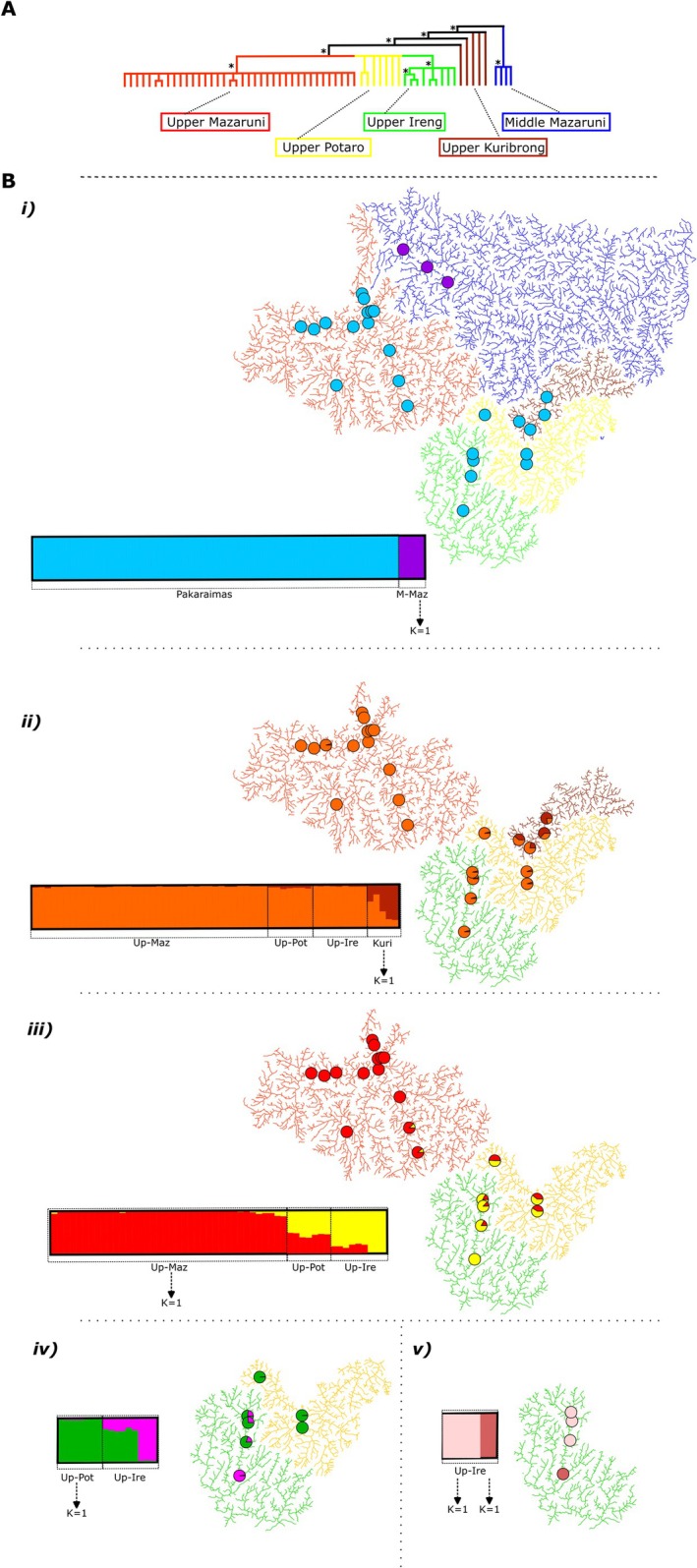
Species‐tree and cluster‐analysis for *Krobia potaroensis* in the Pakaraimas region and middle‐Mazaruni. Colors: red = upper‐Mazaruni, yellow = upper‐Potaro, green = upper‐Ireng, brown = Kuribrong, and blue = middle‐Mazaruni. (A) Simplified SVDQuartets tree showing branches with bootstrap support = 100% (*) and > 70% but < 100%, tree. (B) [i] to [v] results of hierarchical STRUCTURE analyses and corresponding map of sample distributions with: [i] *K. potaroensis* from the rivers of the Pakaraimas and samples from the middle‐Mazaruni (*K* = 2; *n* = 63), [ii] samples from river systems of the Pakaraimas (*K* = 2; *n* = 59), [iii] *K. potaroensis* from the upper‐Mazaruni, upper‐Potaro, and upper‐Ireng (*K* = 2; *n* = 54), [iv] K. potaroensis from the upper‐Potaro and upper‐Ireng (*K* = 2; *n* = 16), and [v] *K. potaroensis* from the upper‐Ireng (*K* = 2; *n* = 9). Maps corresponding to analyses in panel A showing *K. potaroensis* sample locations within the river systems for analyses. Pie‐charts show proportional cluster‐membership for representative samples from each sampling site.

We found strong bootstrap support (> 95%) in the RAxML tree for three broader clades in the analyzed taxa (Figure 2): (i) the chosen outgroup taxa (*Aequidens* and *Cichlasoma*), (ii) the ‘lowland *Krobia*’ group (*K. xinguensis*, *K. paloemeuensis*, *K*. sp. ‘Sinnamary’, *K. itanyi, K. petitella*, and *K. guianensis* ), and (iii) the ‘*potaroensis*‐clade’, formed by all individuals of *K*. sp. ‘Middle Mazaruni’ and *K. potaroensis* (Ireng + Kuribrong + Upper‐Mazaruni + Upper‐Potaro; Figures 1 and 2). *Krobia* sp. ‘Middle Mazaruni’ and *K. potaroensis* in the Pakaraimas river are separated from other *Krobia* by a long shared branch and their monophyly is strongly supported, indicating a closer relationship between these populations than previously known, and strongly suggesting that lowland *Krobia* from the middle Mazaruni River of Guyana do not share a recent common evolutionary and biogeographic history with the rest of the lowland *Krobia* species (*K. guianensis, K. petitella*) (Regan 1905) found throughout lowland Guyana and Suriname (Taphorn et al. 2022). Among samples within the Pakaraimas river systems, only the Ireng River and upper Mazaruni River samples grouped into highly supported clades (100% bootstrap support), both nested within a poorly supported wider clade of upper Potaro and Kuribrong samples (Figure 3), indicating a recent evolutionary relationship between these clades.

#### 3.2.2 Species Tree and Clustering Analyses

Species tree analyses by SVDQuartets (Figures 3A and S1) further supported the Middle Mazaruni *Krobia* (*K*. sp. ‘Middle Mazaruni’) as a separate clade sister to all *K. potaroensis* samples from the rivers of the Pakaraimas highlands. Within *K. potaroensis*, both the *potaroensis*‐clade and Pakaraimas datasets grouped the upper Mazaruni, the upper Ireng, and the upper Potaro rivers as monophyletic with respect to each other and forming a moderately supported (> 70% bootstrap) clade sister to a non‐monophyletic arrangement from the Kuribrong River (Figure 3A). Further subdivision within river systems was not supported by the SVDQuartets tree.

Cluster analyses in *STRUCTURE* recovered each river system as a distinct population (Figure 3). First, the middle Mazaruni River system was determined as distinct relative to the upland Pakaraimas drainages (Figure 3A,B‐i, *K* = 2) with samples in the Pakaraimas sorting entirely with the first cluster (100% cluster 1) and the middle Mazaruni with the second cluster (100% cluster 2). The second *STRUCTURE* run (the Pakaraimas dataset, excluding middle Mazaruni samples; Figure 3B‐ii) separated the Kuribrong relative to the other three Pakaraimas river systems (*K* = 2). In this second run (*n* = 59) the Mazaruni, Ireng, and Potaro rivers clustered almost entirely in group 1 (> 95% cluster 1) while the Kuribrong River individuals were shared among both cluster 1 and cluster 2 (or historical populations); with individuals falling 16%–78% with cluster 1, and 22%–84% with cluster 2. In this second analysis the Potaro River mostly grouped with the Mazaruni and Ireng (> 92% cluster 1), but with a 5%–8% membership in cluster 2 (Figure 3B‐ii). The third *STRUCTURE* run excluded Kuribrong samples (*n* = 54) and again found *K* = 2 to be the most likely number of ancestral populations (Figure 3B‐iii). This third *STRUCTURE* run mostly separated the Mazaruni River (87%–100% cluster 1) from the Ireng and Potaro rivers (50%–100% cluster 2). Additionally, the cluster analyses revealed that the samples from two most upstream Mazaruni sites (UMaz‐12 and UMaz‐13; Table S1) partially grouped with the Potaro and Ireng (11% and 12% cluster 2; Figure 3B‐iii). The final *STRUCTURE* runs then showed an association between the upper Potaro and upper Ireng rivers (Figure 3B‐iv,v, *n* = 16 *n* = 9, respectively); (*K* = 2), with the main genetic break being at the Orinduik Falls, which separate the most downstream (i.e., lower) Ireng samples (*n* = 3) from Ireng and Potaro samples further upstream. The same population substructure was observed in STRUCTURE runs of our alternative SNP‐matrices, demonstrating minimal effect from the missing data between matrices (Figures S2 and S3).

#### 3.2.3 Principal Components Analysis and Measures of Genetic Diversity

**FIGURE 4:**
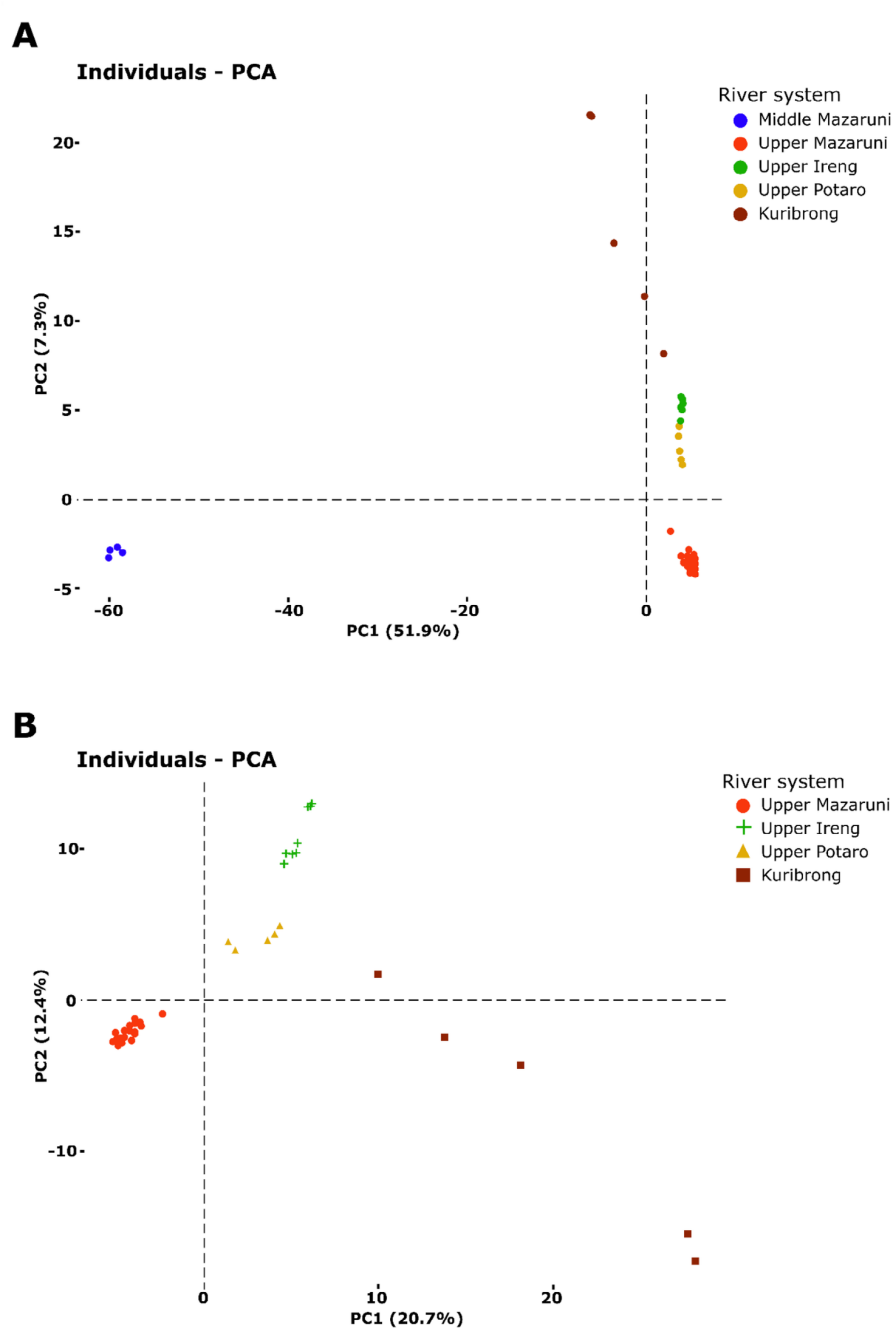
Genetic principal components analysis for (A) the *‘potaroensis*‐clade’ (*n* = 63) and (B) the Pakaraimas‐dataset of *Krobia potaroensis* (*n* = 59).

The principal components analysis (PCA) corroborated the findings of the other analyses in general patterns of inter and intra‐system genetic diversity and population structure. We observed consistent patterns of genetic population structure and diversity between the *potaroensis*‐clade dataset and the three alternative Pakaraimas datasets (Figures 4, S4, and S5). Greater genetic variation between river systems than within river systems is apparent in the case of all drainages except the Kuribrong (Figure 4); despite similar geographic distances between samples within and between river systems (Figure 1).

**FIGURE 5:**
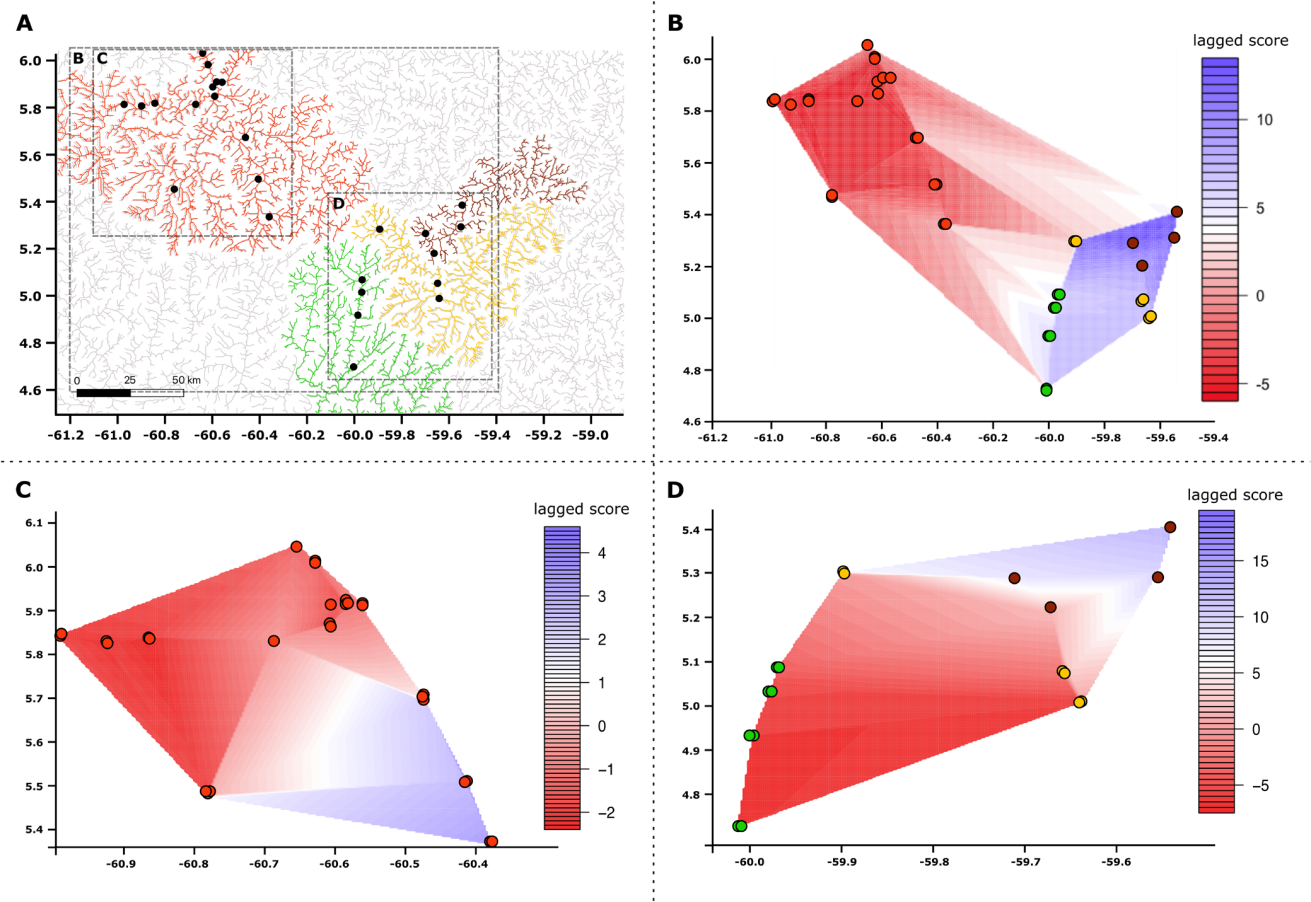
Spatial principal components analysis for the Pakaraimas‐dataset *K. potaroensis*. Panel A representing the four major river systems analyzed: Red = upper Mazaruni River, Green = Upper Ireng River, Yellow = Upper Potaro River, Brown = Upper Kuribrong River. Boxes in panel A correspond with the areas represented in the sPCA plots in panels B–D. Panels B–D: Interpolated maps of sPCA scores with longitude and latitude represented in degrees on the *x* and *y* axes, respectively. Lagged values for principal components are represented as genetic clines along the vectors connecting samples to one another with similar colors representing local genetic structure. Network as determined via calculation of Delauney connection networks.

The spatial principal components analysis (sPCA, Figure 5) allowed for the analyses of principal components while controlling for the distance between samples (calculated via Moran's I). In the sPCA analysis of the entire Pakaraimas dataset, two groups were apparent: one group corresponding to the upper Mazaruni, and the other group corresponding to the Kuribrong, Potaro, and Ireng together (Figure 5B). Additionally, though the sPCA analysis does not model the distance along the river, and instead models a network of linear distances that cross land, the observed relationships between populations were still suggestive of the topography of the region and riverine interdigitation in shaping fish dispersal routes. Interestingly, the two samples in the upper Mazaruni that showed association with the upper Potaro in the *STRUCTURE* analyses again showed association with the other river systems with similar scores to the other (non‐Mazaruni) genetic group along both PC1 and PC2 (Figures 3 and 5). Our subsequent sPCA analyses focused separately on the upper Mazaruni and on the Kuribrong + Potaro + Ireng (Figure 5B,C, respectively). In the analysis of the upper Mazaruni, the most upstream samples are delineated from downstream samples, despite similar lengths of sampled river, highlighting a degree of isolation between upper tributaries and mainstem samples within the upper Mazaruni. Meanwhile, the sPCA of the Kuribrong + Potaro + Ireng rivers again shows a closer association between the Potaro and Ireng river systems, despite the Kuribrong entering the Potaro River downstream of these rivers' respective waterfalls. Measures of observed heterozygosity (*H*_O_) and mean gene diversity (*H*_S_) were moderate for *K. potaroensis* from the upper Mazaruni (*H*_O_ = 0.049; *H*_S_ = 0.051), the upper Ireng (*H*_O_ = 0.045; *H*_S_ = 0.059), slightly higher in the upper Potaro (*H*_O_ = 0.087; *H*_S_ = 0.084), and highest within the Kuribrong (*H*_O_ = 0.17; *H*_S_ = 0.226; Table S3). The relative genetic diversity in the Kuribrong is suggestive of a larger and older population, or of historical connections to other surrounding drainages. Genetic diversity ranged by sample site (Table 2) but did not appear to follow the expected pattern of the downstream‐increase of genetic diversity model (DIGD, Morrissey and De Kerckhove 2009; Paz‐Vinas et al. 2015; Thomaz et al. 2016). Measures of genetic distance were consistent with the population structure observed in the *STRUCTURE* analyses (Table S4). Between river systems *F*_ST_ values ranged from low, around 0.02–0.05 between the upper Ireng and upper Potaro and between the upper Mazaruni and upper Potaro, to high, 0.28–0.32 between the below‐waterfall Ireng site and sites in the upper Mazaruni and Kuribrong (Table S4).

**TABLE 2:** Observed heterozygosity (*H*_O_) at each site in the river systems of the Pakaraimas from upstream (low order tributaries) to more downstream sites.

**Kuribrong upstream**	**Potaro upstream**	**Ireng upstream**	**Mazaruni upstream**
0.0817 (Kuri‐04)	0.078 (Upot‐03)	0.0516 (UIre‐04)	0.0407 (UMaz‐14)
0.1706 (Kuri‐03)	0.0877 (Upot‐02)	0.0432 (UIre‐03)	0.0548 (UMaz‐13)
0.2445 (Kuri‐02)	0.0856 (Upot‐01)	0.0642 (UIre‐02)	0.053 (UMaz‐12)
0.2661 (Kuri‐01)		0.0285 (UIre‐01)	0.056 (UMaz‐11)
			0.0456 (UMaz‐10)
			0.0459 (UMaz‐09)
			0.0511 (UMaz‐08)
			0.0509 (UMaz‐07)
			0.0454 (UMaz‐06)
			0.0533 (UMaz‐05)
			0.0498 (UMaz‐04)
			0.0476 (UMaz‐03)
			0.0421 (UMaz‐02)
			0.0407 (UMaz‐01)
**Kuribrong downstream**	**Potaro downstream**	**Ireng downstream**	**Mazaruni downstream**

*Note:* See Table S1 for GPS of each site. Highest and lowest values within each river system are underlined.

#### 3.2.4 Demographic History Between River Systems

The preferred model for 9 of 10 pairwise population comparisons was the isolation‐with‐migration (IM) model (ΔAIC > 11 in all cases), with only the Middle Mazaruni to Upper Mazaruni comparison favoring a different model (no_mig_size ΔAIC = 74.2 over the next best model; see Tables S5 and S6 for the full model results). Divergence times among the upper Pakaraimas river systems were estimated as relatively recent (< 4 kya in all comparisons), suggesting either very recent isolation, or apparent recency through periodic gene flow. As expected, the Middle Mazaruni diverged substantially earlier in all models (~38 kya—84 kya for all IM models), including an extreme estimate between the Middle‐ and Upper Mazaruni of > 1MY. In this latter case, this estimate also approaches the upper bound of the parameter, and further investigation of this divergence time may reveal this to be a minimum divergence time. Migration rates under the IM model were low to moderate across all pairwise comparisons (migration rate 10^−4^ to 10^−7^ per generation) suggesting that any contemporary dispersal between river systems is rare. We note that while alternative calculations of generation time (e.g., 2–3 years) would shift these estimates proportionally, the divergence times would still be on the scale of ~10^3^ to 10^4^ years in the upland rivers.

## 4 Discussion

### 4.1 *Krobia* in the Uplands and Lowlands of the Guiana Shield

The monophyly of *K. potaroensis* along with the deep branch length that separates this lineage from the rest of the genus (Figure 1) suggests a long period of isolation in the Pakaraima uplands, and a previously undocumented biogeographic relationship between upland *Krobia* and related populations in the surrounding lowland regions of the western Guiana Shield. *Krobia potaroensis*, like many other fishes, is endemic to the Pakaraimas region (Alofs et al. 2014; Hardman et al. 2002; Hayes et al. 2020; López‐Fernández et al. 2012; Maldonado‐Ocampo et al. 2014; Taphorn et al. 2008, 2017). The Miocene‐Pleistocene rearrangements of Guiana Shield paleodrainages, such as the Grand Pakaraima River and the Proto‐Essequibo, likely shaped the region's fish biogeography through geodispersal events (McConnell 1968; Akin et al. 2024; Lujan and Armbruster 2011). The sister relationship between middle‐Mazaruni *Krobia* and those in the Pakaraimas indicates historical upland–lowland connections, possibly linked to these drainage rearrangements. While no divergence dates are currently available to test these hypotheses, the outgroup position and high genetic diversity of Kuribrong River *K. potaroensis*, relative to the other Pakaraimas populations, suggests unrecognized historical conduits to the middle Mazaruni, highlighting the river's potential importance in shaping regional biogeography. Such relationships are akin to those found between the Pakaraimas *Mazarunia* and the lowland *Guianacara* cichlid genera (Ilves et al. 2018; López‐Fernández et al. 2010, 2012). Unlike *Guianacara*, however, the distribution of *Krobia* is not known to extend into the western Guiana Shield, which now drains into the Orinoco River basin. The Pakaraimas clade of *Krobia* is sister to more eastern ‘lowland’ species in the ML‐tree, indicating historical separation between upland and lowland clades and a divergent biogeographic history. This pattern is superficially similar to that observed in the knifefish *Gymnotus carapo*, which has an upland Pakaraimas clade (along with the upper Berbice River), that is sister to lineages found throughout the eastern lowlands of the Guiana Shield (Lehmberg et al. 2018). Unlike *Gymnotus*, however, we found no evidence of common ancestry between the Pakaraimas and the Berbice river endemic *Krobia petitella*. Moreover, in contrast to the long branch lengths we observe between upland and lowland *Krobia*, *G. carapo* showed relatively shallow divergence between upland and lowland populations, possibly linked to long‐distance dispersal and an ability to surpass elevational river barriers (Lehmberg et al. 2018). Contrasting the potentially recent isolation of upland *G. carapo* , the isolation of several endemic catfishes (suborder: Loricariodei (Rafinesque 1815)) above the large waterfalls of the Pakaraimas has been hypothesized to predate the uplift of planation surfaces in the Oligocene (Armbruster and Taphorn 2011; Lujan and Armbruster 2011; Lundberg et al. 2007; Taphorn et al. 2010). Taken together, the modern biogeography of the Pakaraimas reflects repeated isolation of both relictual upland lineages and lowland lineages that more recently expanded into the uplands, likely driven by the region's episodic geologic uplift. The presence of several of these clades in multiple basins across the Pakaraimas highlights that the connections between river systems are either recurring or persistent, thus leading to the observed multi‐river distributions of species that appeared to have arrived in the region at markedly differing times.

Our phylogenetic results suggest that *Krobia* may also serve to reveal wider regional biogeographic patterns. *Krobia* occurs across the Guianas, spanning two potentially distinct eastern versus western biogeographical units (Lemopoulos and Covain 2019). Further, *Krobia* is likely demonstrative of dispersal corridors that led to the high rates of endemism in the western Guiana Shield (Faustino‐Fuster et al. 2021), as well as connections between the eastern Guiana Shield and more southern drainages of the Amazon and the Brazilian Shield (Lemopoulos and Covain 2019). The relationships among lowland species of *Krobia* (Figure 1) suggest associations between the eastern Amazon (*K. xinguensis*), the mid‐elevations of southern Suriname (*K. paloemeuensis*), and the coastal rivers of the Guianas ( *K. guianensis* , *K. petitella*, *K. itanyi* and *K*. sp. *‘*Sinnamary’), all of which warrant further investigation. Thus, *Krobia* is a potential model for testing hypothesized historical connections between the lower Amazon and southern Suriname that appear to have led to sister clade relationships among fishes across the region (e.g., *Corydoras* (Nijssen 1970); *Guyanancistrus* (Fisch‐Muller et al. 2018)).

### 4.2 Genetic Population Structure of *Krobia* in the Pakaraimas Mountains

Out‐of‐network dispersal routes, which are not typically considered in models of riverine connectivity, are apparent in the population structure of *K. potaroensis*. The low levels of genetic differentiation that we observed for *K. potaroensis* between upland interdigitating tributaries of differing river networks are consistent with out‐of‐network connections between all four upland systems (Figures 3, 4, 5). Populations interrupted by larger waterfalls, such as the Kuribrong from the Potaro, and the upper Ireng from the lower Ireng (Figure 3B‐ii,v, respectively), exemplify the effect of large barriers, resulting in greater differentiation within‐network than is observed between basins. Less apparent in our analyses are the effects of smaller barriers, such as rapids and small waterfalls. The interplay between species vagility, the differing hydrology of barriers, and the possibility of unidirectional gene flow in a downstream direction likely create specific conditions for gene flow in different basins and warrant further study (e.g., Tamario et al. 2025).

In our analyses, a similar scale of genetic differentiation is observed, in many cases, between rivers as is seen within rivers. This suggests that dispersal between drainages may be recurring on contemporary timescales. Reinforcing the connections between Pakaraimas rivers, upper Potaro drainage populations were intermediate between the other basins in the analyses both without (*STRUCTURE*, PCA, and *F*_ST_) and with explicit spatial information (sPCA). Given the Potaro's central position, it interdigitates with all three other upland river systems but is longitudinally connected only to the Kuribrong, downstream of the escarpment that transects both east–west drainages. An association between the upper Potaro and upper Ireng rivers is apparent throughout our analyses, suggesting recent or ongoing connections between these two rivers that drain in divergent directions (east and south, respectively) of the Guiana Shield plateau. Moreover, inter‐system dispersal between upland river systems is suggested in our demographic analyses, with predominant support for an isolation‐with‐migration (IM) model with low (but non‐zero) dispersal between river systems (migration rate of 10^−4^ to 10^−7^ per generation). Despite the apparent hydrologic barriers between contemporary rivers, divergence times between river systems are estimated to be relatively recent (~10^3^ to 10^4^ years). These recent estimates could be due to occasional migration between river systems facilitated by ephemeral connections, rather than strict isolation of each drainage. However, we also note that the estimated divergence times in these analyses vary widely, especially in pairwise comparisons that include the middle Mazaruni River.

The hydrological relationships between upland Pakaraimas river systems and their lowland reaches have shifted dramatically through the Neogene and Quaternary (Akin et al. 2024). Consistent with other results, middle Mazaruni *Krobia* are modeled to be the most isolated population in the demographic analysis, relative to the populations in the highland rivers of the Pakaraimas. Nevertheless, the preferred model still incorporates some migration between upland rivers and the middle Mazaruni (though at a very low migration rate of ~10^−6^ to 10^−8^; Table S6). In pairwise comparisons between rivers, our demographic analyses estimate that the middle Mazaruni has been isolated from the upland rivers for much longer (10^4^ to 10^6^ years) than the upland rivers have been isolated from one another (~10^3^ to 10^4^ years). Despite contemporary hydrologic connectivity *Krobia* from the upper and middle Mazaruni show no closer genetic association relative to the other upland systems. Further study of the genetic relationships between fishes in the upland Pakaraima rivers and their downstream congeners could clarify the river‐capture events that shaped the contemporary hydrology of the region. In particular, the hypothesized Pliocene‐Pleistocene dissolution of the north‐flowing Grand Pakaraima River paleodrainage, and the consequent expansion of the proto‐Essequibo to its modern configuration, would have substantially shaped the biogeography of Pakaraima fishes (Akin et al. 2024). The Grand Pakaraima River is hypothesized to have connected upland tributaries of the Potaro to the upper Mazaruni and to have flowed northward without connection to most of the contemporary middle Mazaruni. This major rearrangement of drainages, in relatively recent time, highlights the magnitude of drainage rearrangements that still require clarification in the Guianas. Investigating inter‐basin connections known to facilitate fish dispersal, such as systems with well‐characterized river captures or seasonal floodplains, may also provide illustrative tests of how periodic gene flow influences demographic models for Neotropical fishes in less understood systems such as the Pakaraimas highlands.

Inter‐basin hydrological connections likely structure the populations *of K. potaroensis* and explain its distribution in each of the river systems of the Pakaraimas. The multi‐river distribution of *K. potaroensis* is consistent with recent studies of the knifefish *G. carapo* , as well as *Trichomycterus* and *Brachyglanis* catfishes, each of which has genetic lineages occurring in multiple Pakaraimas river basins (Faustino‐Fuster et al. 2021; Hayes et al. 2020; Lehmberg et al. 2018). Further understanding the abiotic and biotic factors that have led to these species becoming widespread in the region, while a large proportion of other fishes remain endemic to a subset of the rivers, is critical to understanding the evolution of the unique fish diversity of the Pakaraimas, their persistence, and consequently their conservation. Consistent with previous studies, the upper Mazaruni River was highlighted as potentially the most isolated river system draining the Guiana Shield plateau (Figure 5). The relative isolation of the upper Mazaruni may explain the extreme patterns of endemism that characterize its distinct fish fauna; and casts a sobering light on the urgency of enacting conservation measures to protect this distinct riverine community from the threat of expanding industrial and artisanal mining pressure (Alofs et al. 2014; Montaña et al. 2021). Further, considering that the Pakaraimas also harbor many species that are endemic to one, or a subset of the upland rivers, inter‐species comparisons in the Pakaraimas rivers are also a rich source of insight into riverscape genomic mechanisms and should be further investigated to better inform conservation priorities and understand out‐of‐network dispersal processes generally (Alofs et al. 2014; Eigenmann 1912; Hayes et al. 2020; Lehmberg et al. 2018; Taphorn et al. 2017, 2022).

From a broader perspective, we did not observe a pattern of DIGD in populations of *K. potaroensis*, which potentially reinforces the influence of out‐of‐network dispersal in structuring populations across the Pakaraimas. Interconnection between upstream tributaries through out‐of‐network dispersal would violate one of the assumptions of the DIGD model, that upstream tributaries are the most isolated (i.e., least connected) river reaches. The more homogeneous distribution of genetic diversity that we observed in the Pakaraimas rivers may be best modeled as a metapopulation. However, a direct comparison of *K. potaroensis* population structure to the theoretical expectations of riverine metapopulation structure (with out‐of‐network gene flow) requires expansion of riverscape genomic models to further consider river interconnections.

Beyond natural, occasional connectivity, human‐mediated translocations may represent a mechanism of inter‐system dispersal for fishes, and this has been observed for many systems globally (Leprieur et al. 2008). In particular, freshwater systems in the Neotropics have seen fish translocations related to economic activities such as aquaculture, sport‐fishing, and the aquarium trade (Brosse et al. 2021; Carvalho et al. 2009; Gubiani et al. 2018). However, neither our analyses nor repeated field observations (DCT, HLF, NKL, EAL, KMA, DHN) provide any indication of human‐mediated transport for *Krobia* in the upland rivers of the Pakaraimas, which are located in a very remote and scarcely population region of Guyana. Human‐mediated transport, where it has occurred, could represent an interesting comparison case to the natural processes we describe here.

The sampling of Neotropical fish communities comes with many logistical difficulties stemming from factors such as the extreme fish diversity in South America and within subregions of the continent (Birindelli and Sidlauskas 2018; Taphorn et al. 2022), as well as the extreme isolation of many river systems such as those in this study. Indeed, the Kuribrong drainage is accessible by helicopter as, to our knowledge, not even Indigenous tribes permanently inhabit the basin. We leveraged sample sets that were accumulated from multiple expeditions to the Pakaraima Mountains region, with resulting variation in sample size and representing a > 10‐year time period (see Table S1). However, where observable (e.g., between sites sampled in 2008, 2011, and 2019 in the upper Mazaruni), we did not observe any genetic differentiation between temporal cohorts.

Our analyses of *K. potaroensis* demonstrate that genetic population structure does not necessarily follow the network architecture of perennial river system reaches, and that out‐of‐network dispersal has likely occurred recently. Our results support that additional consideration should be given to connections between upland tributaries both within a river network and between distinct, neighboring river systems. Ultimately, models that incorporate these dispersal routes should provide more realistic and useful representations of riverine population structure for many systems than relatively simple DIGD models currently make possible. Not least, beyond illuminating biogeographic and ecological mechanisms structuring riverine biodiversity, such models would introduce powerful new and more nuanced predictions to the toolbox of aquatic conservation and management.

### 4.3 Modeling Riverine Connectivity With Out‐of‐Network Dispersal

The extension of riverine structure models from linear systems to consider a dendritic architecture critically advanced our understanding of the hydrologic connectivity, water chemistry, and riparian zones of river systems (Fagan 2002). Episodic flooding of river systems is understood to be a principal factor governing organic nutrient input (Junk 2001) and lateral connectivity to floodplain habitat, and numerous niches and ecological roles have been characterized for fishes and other aquatic species within ‘intermittent rivers and ephemeral streams’ (IRES) (Hawes and Peres 2014; Messager et al. 2021; Stoffels et al. 2016). Seasonal flooding facilitates fish dispersal throughout much of tropical South America, such as the Rupununi Portal in southern Guyana (De Souza et al. 2012, 2020), the Llanos del Orinoco system in Venezuela and northern Colombia (Layman and Winemiller 2005; Winemiller 1990), the Llanos de Moxos system in Bolivia (Puelles et al. 2016), and the Pantanal system of Brazil (Silveira and Weiss 2014). Further, differing ecologies, vagilities, and life history strategies of fish species have been linked to their distributions along the river network and in the flooded riparian zone, and consequently to their home range sizes (Alp et al. 2012; Burgess et al. 2013; Comte and Olden 2018; Stoffels et al. 2016). Amphibious fishes then further exemplify out‐of‐network dispersal. Species with terrestrial life stages (e.g., Rivulidae (Pace and Gibb 2014)), or tolerance for extended overland movement (e.g., callichthyid and loricariid catfishes (Bressman 2020; Graham 1997)), also represent ecologies that would violate the assumption of within‐network dispersal. Extending studies to characterize the links between species ecology and vagility, their seasonal presence within the flooded riparian environment, and an increased dispersal probability between adjacent river channels is therefore critical to more comprehensive models of riverine population structure and biodiversity.

Conceptual models of riverine population structure require extension to consider IRES and out‐of‐network dispersal of aquatic taxa (Box 1). Existing models of riverine population structure are valuable in providing sets of expectations with which to compare empirical observations. However, these models assume a fixed network architecture for river systems in a context in which researchers increasingly recognize the degree to which river systems are hydrologically variable. IRES are estimated to make up 51%–60% of river basin length and are therefore fundamental to consider when studying river network architecture (Messager et al. 2021). Observed fish movements and ecological functions within IRES and flooded forests demonstrate their role as out‐of‐network dispersal conduits (De Souza et al. 2012, 2020; Goss et al. 2014; Hawes and Peres 2014; Stoffels et al. 2016). Consistent with this, our results show that IRES facilitate gene flow between river basins in the Pakaraimas.

Out‐of‐network dispersal is most obvious when connecting otherwise distinct river systems resulting in the admixture of distinct ancestral populations detectable as distinct clusters. Importantly, the ability to disperse between river systems (Box 1‐D‐I) likely involves the same hydrologic mechanisms (i.e., transient flooding, river avulsion, or river capture) that would allow dispersal and gene flow within a river system (Box 1‐D‐II). The possibility of alternative dispersal routes, beyond the classic longitudinal within‐system dispersal, presents a mechanism by which otherwise isolated upland river systems (e.g., by rapids or small waterfalls) can be reached by more‐downstream populations or by those in adjacent river systems. Violating the assumption that aquatic taxa are confined to the perennial river channel, out‐of‐network dispersal may complicate the validity, or at least the interpretation, of measures of isolation‐by‐distance (Wright 1943) and isolation‐by‐resistance (McRae 2006). Furthermore, by challenging assumptions about the isolation of upstream tributaries, instances of gene flow between adjacent (or interdigitating) rivers may enhance the stability and long‐term persistence of upland populations (Csermely 2004; Lowe 2002; Tamario et al. 2025; Whiteley et al. 2015), while also contributing to higher beta‐diversity and overall patterns of biodiversity observed in upland systems (Finn et al. 2011; Lujan and Conway 2015).

In Box 1, we highlight the standard expectations of the DIGD model of riverine populations, that is, greater genetic diversity downstream and greater genetic differentiation upstream (Morrissey and De Kerckhove 2009; Paz‐Vinas and Blanchet 2015; Thomaz et al. 2016), and further extend the model to include out‐of‐network dispersal. Existing models of riverine structure, such as DIGD, the Stream Hierarchy Model, and the Death Valley Models retain substantial value in describing the observed population structure of many fish species when out‐of‐network dispersal can be reasonably discounted due to hydrologic or ecologic factors (Blanchet et al. 2020; Comte and Olden 2018; Meffe and Vrijenhoek 1988; Morrissey and De Kerckhove 2009; Piller et al. 2024). The discussion of out‐of‐network dispersal has developed in consideration of riverine taxa that have terrestrial or aerial life stages, such as amphibians (Campbell Grant et al. 2007, 2010; Rissler et al. 2003) and insects (Bogan and Boersma 2012; Finn et al. 2007; Lancaster et al. 2024; Macneale et al. 2005), and led to models such as the Headwater Model (Finn et al. 2007) and the Widespread Gene Flow Model (Hughes et al. 2009), but those models do not realistically depict the dynamics of water‐bound organisms such as fishes. Extending those models, we highlight that assessing the population structure of aquatic taxa needs to also consider hydrologic out‐of‐network dispersal routes because freshwater out‐of‐network corridors differ from terrestrial routes in their permeability and their temporal persistence. Extensions to riverscape models to incorporate out‐of‐network dispersal should be considered in regions where the hydrology of the region and the vagility of aquatic taxa may facilitate these alternative dispersal routes. Further investigation of freshwater out‐of‐network corridors needs to consider the hydrology and architecture of river systems and how it translates to their use by water‐bound species of differing ecologies and vagilities.

## 5 Conclusion

The inter‐basin connections we characterized in the Pakaraima Mountains of the Guiana Shield of South America have both regional conservation value and broader implications for the modeling of riverine population structure. Understanding the historical biogeography of freshwater fishes and other riverine organisms, as well as their conservation, in the Pakaraimas rivers and elsewhere, depend on the accurate characterizations of their evolutionary history, life history attributes, and resilience to anthropogenic disturbance. River systems interdigitate in uplands throughout the Neotropics and other mountainous regions around the world, thus characterizing the role of ephemeral connections is crucial to a comprehensive perspective of how riverine aquatic populations and communities are distributed in unique network‐structured landscapes. A synthesis of models of contemporary riverine population structure with models of historical biogeography and hydrogeology are critical in understanding the interplay between contemporary population structure, evolutionary processes, and historical biogeography.

## Author Contributions

**Thomas D. Morgan:** conceptualization (lead), data curation (lead), formal analysis (lead), investigation (lead), methodology (lead), project administration (lead), writing – original draft (lead), writing – review and editing (equal). **Karen M. Alofs:** data curation (supporting), funding acquisition (supporting), supervision (supporting), writing – original draft (supporting), writing – review and editing (equal). **Donald C. Taphorn:** data curation (supporting), investigation (supporting), validation (supporting), writing – original draft (supporting), writing – review and editing (equal). **Devya Hemraj‐Naraine:** data curation (supporting), investigation (supporting), writing – original draft (supporting), writing – review and editing (equal). **Elford Liverpool:** data curation (supporting), investigation (supporting), project administration (supporting), resources (supporting), writing – original draft (supporting), writing – review and editing (equal). **Jan Mol:** data curation (supporting), resources (supporting), validation (supporting), writing – original draft (supporting), writing – review and editing (equal). **Raphael Covain:** data curation (supporting), writing – original draft (supporting), writing – review and editing (equal). **Nathan K. Lujan:** data curation (supporting), methodology (supporting), writing – original draft (supporting), writing – review and editing (equal). **Hernán López‐Fernández:** conceptualization (lead), data curation (equal), funding acquisition (lead), investigation (equal), methodology (equal), project administration (lead), resources (lead), supervision (lead), validation (equal), writing – original draft (equal), writing – review and editing (equal).

## Funding

This work was funded by Discovery Grants from the Natural Sciences and Engineering Research Council of Canada, the Royal Ontario Museum, Toronto, Canada, World Wildlife Fund (WWF)‐Guianas, and the University of Michigan, and through block grants from the University of Michigan Museum of Zoology.

## Conflicts of Interest

The authors declare no conflicts of interest.

## Supporting information


**Table S1:** Museum collection information for *Krobia potaroensis* and selected outgroups used in phylogenetic and population genetic analyses. **Two samples were originally identified in the field as being from Kuribrong tributaries. Subsequent analysis of their GPS with detailed hydrological maps places them in the Potaro, the original naming convention (‘Kuri’) is reflected in the raw sequence files. ***Kr‐pot‐maz‐1 sample had uncertain GPS coordinates within the upper Mazaruni, and was therefore excluded from site‐specific analyses (sPCA, genetic distance and genetic diversity measures). Museum abbreviations: AUM = Auburn University Museum, ROM = Royal Ontario Museum, UMMZ = University of Michigan Museum of Zoology, RP = Redpath Museum—McGill University. LBP = Laboratório de Biologia e Genética de Peixes, Universidade Estadual Paulista “Júlio de Mesquita Filho”, Sao Paulo, MHNG = Muséum d'Histoire Naturelle de la Ville de Genève.
**Table S2:** Sequence and exported‐matrix information for ddRAD library of *Krobia potaroensis* in the Pakaraima Mountains region of western Guyana.
**Table S3:** Observed heterozygosity (*H*_O_) at each site in the river systems of the Pakaraimas from upstream (low order tributaries) to more downstream sites. See Table S1 for GPS sites of each site. Lowest and highest values within each river system are identified with * and ** respectively.
**Table S4:** Genetic distance, Hudson's (*F*_ST_) for *Krobia potaroensis* between sampling sites in the Pakaraima Mountains of western Guyana (see Table S1) based on 11,173 biallelic SNPs. Brown = the Kuribrong River, green = the upper Ireng River, red = the upper Mazaruni River, and yellow = the upper Potaro River. Within‐river genetic distances are bordered by a single line, while between‐river genetic distances are bordered with a double line.
**Figure S1:** SVDQuartets tree (Swofford 2002; Chifman and Kubatko 2014) for *Krobia potaroensis* individuals in the Pakaraima Mountains of western Guyana. Sample names are as in Table S1. Tree was generated using a matrix of 2,975,799 bp from 10,366 loci. Robustness of relationships at each node was assessed through 1000 bootstrap replicates.
**Figure S2:** STRUCTURE plots for the 6516 unlinked SNPs for loci present in at least 15 of 59 *Krobia potaroensis* (*n* = 59) in the Pakaraima Rivers of western Guyana. Following a burn‐in of 100,000 MCMC iterations each STRUCTURE (Pritchard et al. 2000) run was conducted with 1000,000 MCMC iterations with 10 independent searches of each *K* value ranging from 1 to 7; parallelized using Strauto (v1.0 Chhatre and Emerson 2017). Convergence was assessed with Structure Harvester (Earl and vonHoldt 2012) and results were summarized using the Clumpak pipeline (Jakobsson and Rosenberg 2007; Kopelman et al. 2015). Cluster assignments are shown for values of A: *K* = 2, B: *K* = 3, and C: *K* = 4. Sample order: (i) lower Ireng River (*N* = 3), (ii) upper Ireng River (*N* = 5), (iii + vi) upper Potaro River (*N* = 2 and 5 respectively), and (v) the upper Mazaruni River (*n* = 38).
**Figure S3:** STRUCTURE plots for the 5895 unlinked SNPs for loci present in at least 30 of 59 *Krobia potaroensis* (*n* = 59) in the Pakaraima Rivers of western Guyana. Following a burn‐in of 100,000 MCMC iterations each STRUCTURE (Pritchard et al. 2000) run was conducted with 1000,000 MCMC iterations with 10 independent searches of each *K* value ranging from 1 to 7; parallelized using Strauto (v1.0 Chhatre and Emerson 2017). Convergence was assessed with Structure Harvester (Earl and vonHoldt 2012) and results were summarized using the Clumpak pipeline (Jakobsson and Rosenberg 2007; Kopelman et al. 2015). Cluster assignments are shown for values of (A) *K* = 2, (B) *K* = 3, and (C) *K* = 4. Sample order: (i) lower Ireng River (*N* = 3), (ii) upper Ireng River (*N* = 5), (iii + vi) upper Potaro River (*N* = 2 and 5 respectively), and (v) the upper Mazaruni River (*n* = 38).
**Figure S4:** Genetic principal components analysis for *Krobia potaroensis* (*n* = 59) from the upland rivers of the Pakaraimas Mountains in western Guyana. Analysis conducted in adegenet v2.1.10 (Jombart and Ahmed 2011) for 6515 unlinked SNPs with 15.8% missing data. River systems are abbreviated: UIre = Upper Ireng River, UPot = Upper Potaro River, Kuri = Kuribrong River, and UMaz = Upper Mazaruni River.
**Figure S5:** Genetic principal components analysis for *Krobia potaroensis* (*n* = 59) from the upland rivers of the Pakaraimas Mountains in western Guyana. Analysis conducted in adegenet v2.1.10 (Jombart and Ahmed 2011) for 5895 unlinked SNPs with 10.8% missing data. River systems are abbreviated: UIre = Upper Ireng River, UPot = Upper Potaro River, Kuri = Kuribrong River, and UMaz = Upper Mazaruni River.
**Table S5:** Model comparison in *dadi‐cli* (Gutenkunst et al. 2009; Huang et al. 2021) for six demographic models (Portik et al. 2017) describing the pairwise genetic relationships between five populations of *Krobia potaroensis* in the Pakaraimas of western Guyana. The preferred model (lowest AIC) is listed first for each pair and shown in bold. ΔAIC is relative to the best‐supported model (reference = 0.00). Support ratings: *** = ΔAIC > 10 for all alternatives (strong support); * = marginal support (ΔAIC < 10 for ≥ 1 alternative). ^†^Ireng–Middle‐Maz: sec_contact_asym_mig (ΔAIC = 2.26) and no_mig_size (ΔAIC = 3.63) are close competitors; interpret IM parameters with caution.
**Table S6:** Demographic parameters for preferred models. Best fit parameter estimates, and 95% confidence intervals obtained via parametric bootstrapping in dadi‐cli. Five populations of *Krobia potaroensis* from the Pakaraimas Rivers (upper Mazaruni, upper Potaro River, Upper Ireng River, and Kuribrong River) and the middle Mazaruni River (*n* = 63). For IM (isolation‐with‐migration) models: s = ancestral population size fraction retained by Ireng at split; nu1, nu2 = relative sizes of daughter populations; T = divergence time (years, assuming generation time and mutation rate as described in Methods); m12, m21 = migration rates (proportion of population replaced per generation). For no_mig_size (Upper‐Maz–Middle‐Maz): nu1a, nu2a = initial sizes post‐split; nu1b, nu2b = final sizes; T1, T2 = durations of first and second epochs (years).

## Data Availability

The data underlying this study are available at: https://doi.org/10.5061/dryad.jwstqjqpc.
