# Supplementary material for: Dispersal Across Headwaters Determines Fish Population Structure Between Interdigitating River Systems in the Guiana Shield Highlands
Source: Ecol Evol. 2026 May 21;16(5):e73603. doi: 10.1002/ece3.73603 (PMC13239270; doi:10.1002/ece3.73603)
Supplement: Supplementary file 1 — Table S1: Museum collection information for Krobia potaroensis and selected outgroups used in phylogenetic and population genetic analyses. **Two samples were originally identified in the field as being from Kuribrong tributaries. Subsequent analysis of their GPS with detailed hydrological maps places them in the Potaro, the original naming convention (‘Kuri’) is reflected in the raw sequence files. ***Kr‐pot‐maz‐1 sample had uncertain GPS coordinates within the upper Mazaruni, and was therefore excluded from site‐specific analyses (sPCA, genetic distance and genetic diversity measures). Museum abbreviations: AUM = Auburn University Museum, ROM = Royal Ontario Museum, UMMZ = University of Michigan Museum of Zoology, RP = Redpath Museum—McGill University. LBP = Laboratório de Biologia e Genética de Peixes, Universidade Estadual Paulista “Júlio de Mesquita Filho”, Sao Paulo, MHNG = Muséum d'Histoire Naturelle de la Ville de Genève. Table S2: Sequence and exported‐matrix information for ddRAD library of Krobia potaroensis in the Pakaraima Mountains region of western Guyana. Table S3: Observed heterozygosity (H O) at each site in the river systems of the Pakaraimas from upstream (low order tributaries) to more downstream sites. See Table S1 for GPS sites of each site. Lowest and highest values within each river system are identified with * and ** respectively. Table S4: Genetic distance, Hudson's (F ST) for Krobia potaroensis between sampling sites in the Pakaraima Mountains of western Guyana (see Table S1) based on 11,173 biallelic SNPs. Brown = the Kuribrong River, green = the upper Ireng River, red = the upper Mazaruni River, and yellow = the upper Potaro River. Within‐river genetic distances are bordered by a single line, while between‐river genetic distances are bordered with a double line. Figure S1: SVDQuartets tree (Swofford 2002; Chifman and Kubatko 2014) for Krobia potaroensis individuals in the Pakaraima Mountains of western Guyana. Sample names are as in Tab [file ECE3-16-e73603-s001.docx]

Supplemental Figures and Tables – Pakaraimas population structure interdigitating rivers.

Table S13: Museum collection information for *Krobia potaroensis* and selected outgroups used in phylogenetic and population genetic analyses. **Two samples were originally identified in the field as being from Kuribrong tributaries. Subsequent analysis of their GPS with detailed hydrological maps places them in the Potaro, the original naming convention (‘Kuri’) is reflected in the raw sequence files. ***Kr-pot-maz-1 sample had uncertain GPS coordinates within the upper Mazaruni, and was therefore excluded from site-specific analyses (sPCA, genetic distance and genetic diversity measures). Museum abbreviations: AUM=Auburn University Museum, ROM=Royal Ontario Museum, UMMZ = University of Michigan Museum of Zoology, RP = Redpath Museum – McGill University. LBP = Laboratório de Biologia e Genética de Peixes, Universidade Estadual Paulista “Júlio de Mesquita Filho”, Sao Paulo, MHNG = Muséum d'Histoire Naturelle de la Ville de Genève.

| MUSEUM COLLECTION | TISSUE SAMPLE NUMBER | SPECIES | RIVER SYSTEM - Site ID | LATITUDE | LONGITUDE | SAMPLE NAME IN ANALYSES |
| --- | --- | --- | --- | --- | --- | --- |
| AUM | 6533 | Krobia potaroensis | Upper Potaro River - UPot-03 | 5.30 | -59.90 | Kr-pot-kuri-1** |
| AUM | 6534 | Krobia potaroensis | Upper Potaro River - UPot-03 | 5.30 | -59.90 | Kr-pot-kuri-2** |
| AUM | 10108 | Krobia potaroensis | Upper Ireng River - UIre-01 | 4.73 | -60.01 | Kr-pot-ire-1 |
| AUM | 10109 | Krobia potaroensis | Upper Ireng River - UIre-01 | 4.73 | -60.01 | Kr-pot-ire-2 |
| AUM | 10110 | Krobia potaroensis | Upper Ireng River - UIre-01 | 4.73 | -60.01 | Kr-pot-ire-3 |
| ROM | T21920 | Krobia sp. 'Middle Mazaruni' | Middle Mazaruni | 6.30 | -60.37 | Kr-gui-maz-1 |
| ROM | T21858 | Krobia sp. 'Middle Mazaruni' | Middle Mazaruni | 6.11 | -60.11 | Kr-gui-maz-2 |
| ROM | T21865 | Krobia sp. 'Middle Mazaruni' | Middle Mazaruni | 6.11 | -60.11 | Kr-gui-maz-3 |
| ROM | T21452 | Krobia sp. 'Middle Mazaruni' | Middle Mazaruni. | 6.21 | -60.23 | Kr-gui-maz-4 |
| ROM | T06031 | Krobia potaroensis | Upper Mazaruni | *uncertain GPS | | Kr-pot-maz-1 |
| ROM | T06132 | Krobia potaroensis | Upper Mazaruni (main channel) - UMaz-11 | 5.69 | -60.47 | Kr-pot-maz-2 |
| ROM | T06133 | Krobia potaroensis | Upper Mazaruni (main channel) - UMaz-11 | 5.69 | -60.47 | Kr-pot-maz-3 |
| ROM | T06017 | Krobia potaroensis | Upper Mazaruni (Kukui River) - UMaz-12 | 5.51 | -60.41 | Kr-pot-maz-4 |
| ROM | T06018 | Krobia potaroensis | Upper Mazaruni (Kukui River) - UMaz-12 | 5.51 | -60.41 | Kr-pot-maz-5 |
| ROM | T06055 | Krobia potaroensis | Upper Mazaruni (Kukui River) - UMaz-13 | 5.36 | -60.37 | Kr-pot-maz-6 |
| ROM | T14541 | Krobia potaroensis | Kuribrong River - Kuri-02 | 5.31 | -59.55 | Kr-pot-kuri-3 |
| ROM | T17202 | Krobia potaroensis | Upper Potaro River - UPot-02 | 5.07 | -59.65 | Kr-pot-pot-1 |
| ROM | T17203 | Krobia potaroensis | Upper Potaro River - UPot-02 | 5.07 | -59.65 | Kr-pot-pot-2 |
| ROM | T17204 | Krobia potaroensis | Upper Potaro River - UPot-02 | 5.07 | -59.65 | Kr-pot-pot-3 |
| AUM | 10283 | Krobia potaroensis | Upper Ireng River - UIre-04 | 5.09 | -59.97 | Kr-pot-ire-4 |
| ROM | T17134 | Krobia potaroensis | Upper Potaro River - UPot-01 | 5.01 | -59.64 | Kr-pot-pot-4 |
| ROM | T17135 | Krobia potaroensis | Upper Potaro River - UPot-01 | 5.01 | -59.64 | Kr-pot-pot-5 |
| AUM | 10330 | Krobia potaroensis | Upper Ireng River - UIre-02 | 4.93 | -60.00 | Kr-pot-ire-5 |
| AUM | 10132 | Krobia potaroensis | Upper Ireng River - UIre-03 | 5.04 | -59.98 | Kr-pot-ire-6 |
| AUM | 10286 | Krobia potaroensis | Upper Ireng River - UIre-04 | 5.09 | -59.97 | Kr-pot-ire-7 |
| AUM | 10324 | Krobia potaroensis | Upper Ireng River - UIre-02 | 4.93 | -60.00 | Kr-pot-ire-8 |
| ROM | T06221 | Krobia potaroensis | Upper Mazaruni (Membaru Creek) - UMaz-03 | 5.93 | -60.59 | Kr-pot-maz-7 |
| ROM | T06222 | Krobia potaroensis | Upper Mazaruni (Membaru Creek) - UMaz-03 | 5.93 | -60.59 | Kr-pot-maz-8 |
| ROM | T14570 | Krobia potaroensis | Kuribrong River (Grass Falls Creek Potaro) - Kuri-01 | 5.41 | -59.54 | Kr-pot-kuri-5 |
| ROM | T17233 | Krobia potaroensis | Kuribrong River - Kuri-03 | 5.28 | -59.70 | Kr-pot-kuri-6 |
| ROM | T17295 | Krobia potaroensis | Kuribrong River - Kuri-04 | 5.21 | -59.67 | Kr-pot-kuri-7 |
| UMMZ | T01366 | Krobia potaroensis | Upper Mazaruni - UMaz-06 | 5.87 | -60.61 | Kr-pot-maz-10 |
| UMMZ | T01394 | Krobia potaroensis | Upper Mazaruni - UMaz-08 | 5.84 | -60.87 | Kr-pot-maz-9 |
| UMMZ | T01393 | Krobia potaroensis | Upper Mazaruni - UMaz-08 | 5.84 | -60.87 | Kr-pot-maz-11 |
| UMMZ | T01638 | Krobia potaroensis | Upper Mazaruni - UMaz-02 | 6.00 | -60.63 | Kr-pot-maz-12 |
| UMMZ | T01365 | Krobia potaroensis | Upper Mazaruni - UMaz-06 | 5.87 | -60.61 | Kr-pot-maz-13 |
| ROM | T06160 | Krobia potaroensis | Upper Mazaruni (Waruma Creek) - UMaz-14 | 5.48 | -60.78 | Kr-pot-maz-18 |
| ROM | T06161 | Krobia potaroensis | Upper Mazaruni (Waruma Creek) - UMaz-14 | 5.48 | -60.78 | Kr-pot-maz-16 |
| ROM | T06162 | Krobia potaroensis | Upper Mazaruni (Waruma Creek) - UMaz-14 | 5.48 | -60.78 | Kr-pot-maz-14 |
| ROM | T06163 | Krobia potaroensis | Upper Mazaruni (Waruma Creek) - UMaz-14 | 5.48 | -60.78 | Kr-pot-maz-17 |
| ROM | T06164 | Krobia potaroensis | Upper Mazaruni (Waruma Creek) - UMaz-14 | 5.48 | -60.78 | Kr-pot-maz-15 |
| ROM | T06216 | Krobia potaroensis | Upper Mazaruni (Membaru Creek) - UMaz-03 | 5.93 | -60.59 | Kr-pot-maz-21 |
| ROM | T06217 | Krobia potaroensis | Upper Mazaruni (Membaru Creek) - UMaz-03 | 5.93 | -60.59 | Kr-pot-maz-19 |
| ROM | T06218 | Krobia potaroensis | Upper Mazaruni (Membaru Creek) - UMaz-03 | 5.93 | -60.59 | Kr-pot-maz-20 |
| ROM | T06219 | Krobia potaroensis | Upper Mazaruni (Membaru Creek) - UMaz-03 | 5.93 | -60.59 | Kr-pot-maz-22 |
| ROM | T06027 | Krobia potaroensis | Upper Mazaruni (Kukui River) - UMaz-12 | 5.51 | -60.41 | Kr-pot-maz-23 |
| ROM | T06056 | Krobia potaroensis | Upper Mazaruni (Kukui River) - UMaz-13 | 5.36 | -60.37 | Kr-pot-maz-24 |
| ROM | T06057 | Krobia potaroensis | Upper Mazaruni (Kukui River) - UMaz-13 | 5.36 | -60.37 | Kr-pot-maz-25 |
| ROM | T06029 | Krobia potaroensis | Upper Mazaruni (Kukui River) - UMaz-12 | 5.51 | -60.41 | Kr-pot-maz-26 |
| ROM | T14569 | Krobia potaroensis | Kuribrong River (Grass Falls Creek Potaro) - Kuri-01 | 5.41 | -59.54 | Kr-pot-kuri-8 |
| AUM | 10133 | Krobia potaroensis | Upper Ireng River - UIre-03 | 5.04 | -59.98 | Kr-pot-ire-9 |
| UMMZ | T01546 | Krobia potaroensis | Upper Mazaruni - UMaz-04 | 5.93 | -60.57 | Kr-pot-maz-28 |
| UMMZ | T01547 | Krobia potaroensis | Upper Mazaruni - UMaz-04 | 5.93 | -60.57 | Kr-pot-maz-32 |
| UMMZ | T01653 | Krobia potaroensis | Upper Mazaruni - UMaz-01 | 6.05 | -60.65 | Kr-pot-maz-33 |
| UMMZ | T01507 | Krobia potaroensis | Upper Mazaruni - UMaz-09 | 5.83 | -60.93 | Kr-pot-maz-36 |
| UMMZ | T01508 | Krobia potaroensis | Upper Mazaruni - UMaz-09 | 5.83 | -60.93 | Kr-pot-maz-34 |
| ROM | T06028 | Krobia potaroensis | Upper Mazaruni (Kukui River) - UMaz-12 | 5.51 | -60.41 | Kr-pot-maz-37 |
| UMMZ | T01685 | Krobia potaroensis | Upper Mazaruni - UMaz-05 | 5.92 | -60.61 | Kr-pot-maz-38 |
| UMMZ | T01384 | Krobia potaroensis | Upper Mazaruni - UMaz-07 | 5.83 | -60.69 | Kr-pot-maz-30 |
| UMMZ | T01492 | Krobia potaroensis | Upper Mazaruni - UMaz-10 | 5.84 | -60.99 | Kr-pot-maz-31 |
| UMMZ | T01493 | Krobia potaroensis | Upper Mazaruni - UMaz-10 | 5.84 | -60.99 | Kr-pot-maz-29 |
| UMMZ | T01494 | Krobia potaroensis | Upper Mazaruni - UMaz-10 | 5.84 | -60.99 | Kr-pot-maz-35 |
| UMMZ | T01637 | Krobia potaroensis | Upper Mazaruni - UMaz-02 | 6.00 | -60.63 | Kr-pot-maz-27 |
| UMMZ | T01319 | Krobia paloemeunsis | Palomeu River |  |  | Kr-palo-1 |
| Redpath Museum (Mcgill University) | KX01 | Krobia xinguensis | Xingu River | Aquarium trade |  | Kr-xin-1 |
| ROM | T15315 | *Aequidens tetramerus* | Rio Novo | -4.47 | -53.67 | Ae-tet-nov-1 |
| ROM | T15316 | *Aequidens tetramerus* | Rio Novo | -4.47 | -53.67 | Ae-tet-nov-2 |
| LBP | 16016 | *Aequidens michaeli* | Xingu River | -13.46 | -53.16 | Ae-sp-xin-1 |
| LBP | 16019 | *Aequidens michaeli* | Xingu River | -13.46 | -53.15 | Ae-sp-xin-2 |
| ROM | T20770 | *Cichlasoma bimaculatum* | Demerara River | 6.39 | -58.24 | Cich-bim-1 |
| ROM | T20788 | *Cichlasoma bimaculatum* | Demerara River | 6.35 | -58.24 | Cich-bim-2 |
| ROM | T07815 | *Krobia petitella* | Berbice River | 5.17 | -58.16 | Kr-pett-berb-1 |
| ROM | T08161 | *Krobia petitella* | Berbice River | 5.15 | -58.20 | Kr-pett-berb-2 |
| UMMZ | T00432 | *Krobia guianensis* | Suriname River | 4.51 | -55.33 | Kr-gui-sur-1 |
| UMMZ | T00457 | *Krobia guianensis* | Suriname River | Apresina Rapids |  | Kr-gui-sur-2 |
| ROM | T18846 | *Krobia itanyi* | Maroni/Marowijne River | 5.06 | -54.42 | Kr-ita-mar-1 |
| UMMZ | T02192 | *Krobia itanyi* | Maroni/Marowijne River (Tapanahony) | 2.66 | -55.88 | Kr-ita-mar-4 |
| GFSU | GFSU12-043 | *Krobia guianensis sp. 1 (aff)* | Sinnamary River | 5.31 | -53.05 | Kr-gui1-sinn-1 |
| GFSU | GFSU12-044 | *Krobia guianensis sp. 1 (aff)* | Sinnamary River | 5.31 | -53.05 | Kr-gui1-sinn-2 |

Table S24: Sequence and exported-matrix information for ddRAD library of *Krobia potaroensis* in the Pakaraima Mountains region of western Guyana.

| Pakaraimas *Krobia* raw reads | RAW sequences | loci in 40of79 matrix, of 7440 loci | Loci in Potaroensis-clade-matrix, (of 10,366 loci) | Loci in Pakaraimas-matrix, (of 10,719 loci) |
| --- | --- | --- | --- | --- |
| Kr-pot-ire-1 | 6,378,120 | 7,056 | 9,990 | 10,328 |
| Kr-pot-ire-2 | 3,645,888 | 6,883 | 9,857 | 10,180 |
| Kr-pot-ire-3 | 5,124,906 | 6,815 | 9,931 | 10,267 |
| Kr-pot-ire-4 | 5,922,926 | 7,195 | 10,116 | 10,454 |
| Kr-pot-ire-5 | 5,216,391 | 7,111 | 10,045 | 10,378 |
| Kr-pot-ire-6 | 6,268,663 | 7,182 | 10,144 | 10,477 |
| Kr-pot-ire-7 | 6,594,801 | 7,202 | 10,114 | 10,453 |
| Kr-pot-ire-8 | 4,878,220 | 7,072 | 10,057 | 10,388 |
| Kr-pot-ire-9 | 3,940,074 | 7,224 | 10,207 | 10,544 |
| Kr-pot-kuri-1 | 6,609,263 | 6,838 | 9,825 | 10,161 |
| Kr-pot-kuri-2 | 5,734,180 | 6,961 | 9,929 | 10,251 |
| Kr-pot-kuri-3 | 5,130,148 | 6,540 | 9,702 | 10,009 |
| Kr-pot-kuri-5 | 4,082,697 | 6,976 | 9,894 | 10,205 |
| Kr-pot-kuri-6 | 6,447,444 | 7,113 | 10,021 | 10,354 |
| Kr-pot-kuri-7 | 4,526,840 | 7,091 | 9,962 | 10,291 |
| Kr-pot-kuri-8 | 5,436,436 | 6,699 | 9,882 | 10,194 |
| Kr-pot-maz-1 | 4,391,940 | 6,460 | 9,553 | 9,876 |
| Kr-pot-maz-10 | 2,734,302 | 6,625 | 9,752 | 10,078 |
| Kr-pot-maz-11 | 296,963 | 3,301 | 5,001 | 5,136 |
| Kr-pot-maz-12 | 4,942,429 | 7,050 | 10,151 | 10,492 |
| Kr-pot-maz-13 | 4,000,596 | 6,830 | 9,993 | 10,332 |
| Kr-pot-maz-14 | 9,112,588 | 7,211 | 10,142 | 10,478 |
| Kr-pot-maz-15 | 8,607,287 | 7,216 | 10,108 | 10,445 |
| Kr-pot-maz-16 | 5,937,153 | 7,118 | 9,974 | 10,312 |
| Kr-pot-maz-17 | 4,313,542 | 7,013 | 9,988 | 10,320 |
| Kr-pot-maz-18 | 7,333,518 | 7,106 | 9,994 | 10,325 |
| Kr-pot-maz-19 | 1,264,601 | 5,558 | 8,511 | 8,793 |
| Kr-pot-maz-2 | 4,045,943 | 6,588 | 9,687 | 10,006 |
| Kr-pot-maz-20 | 2,282,707 | 6,512 | 9,641 | 9,956 |
| Kr-pot-maz-21 | 4,074,678 | 7,049 | 10,150 | 10,495 |
| Kr-pot-maz-22 | 3,679,880 | 6,874 | 9,972 | 10,315 |
| Kr-pot-maz-23 | 4,778,490 | 7,012 | 10,140 | 10,494 |
| Kr-pot-maz-24 | 3,113,271 | 6,567 | 9,786 | 10,114 |
| Kr-pot-maz-25 | 4,435,093 | 6,920 | 10,087 | 10,420 |
| Kr-pot-maz-26 | 1,952,138 | 6,490 | 9,658 | 9,969 |
| Kr-pot-maz-27 | 2,481,378 | 6,822 | 10,020 | 10,358 |
| Kr-pot-maz-28 | 3,646,134 | 7,163 | 10,227 | 10,570 |
| Kr-pot-maz-29 | 3,951,534 | 7,206 | 10,235 | 10,584 |
| Kr-pot-maz-3 | 4,422,300 | 6,448 | 9,656 | 9,969 |
| Kr-pot-maz-30 | 3,755,238 | 7,172 | 10,236 | 10,583 |
| Kr-pot-maz-31 | 2,333,245 | 6,850 | 10,040 | 10,371 |
| Kr-pot-maz-32 | 2,953,041 | 6,925 | 10,111 | 10,451 |
| Kr-pot-maz-33 | 3,044,090 | 7,009 | 10,171 | 10,516 |
| Kr-pot-maz-34 | 3,636,834 | 7,077 | 10,212 | 10,552 |
| Kr-pot-maz-35 | 3,075,871 | 6,934 | 10,123 | 10,456 |
| Kr-pot-maz-36 | 2,044,799 | 6,555 | 9,758 | 10,077 |
| Kr-pot-maz-37 | 2,746,900 | 7,008 | 10,142 | 10,481 |
| Kr-pot-maz-38 | 843,605 | 4,975 | 7,690 | 7,922 |
| Kr-pot-maz-4 | 3,708,270 | 6,422 | 9,555 | 9,869 |
| Kr-pot-maz-5 | 6,138,830 | 7,029 | 10,052 | 10,384 |
| Kr-pot-maz-6 | 6,086,339 | 6,976 | 9,961 | 10,302 |
| Kr-pot-maz-7 | 5,006,611 | 7,175 | 10,119 | 10,461 |
| Kr-pot-maz-8 | 7,003,216 | 7,154 | 10,146 | 10,487 |
| Kr-pot-maz-9 | 4,723,318 | 6,967 | 10,113 | 10,447 |
| Kr-pot-pot-1 | 4,894,502 | 6,984 | 9,991 | 10,327 |
| Kr-pot-pot-2 | 4,928,987 | 7,177 | 10,095 | 10,432 |
| Kr-pot-pot-3 | 5,915,726 | 7,200 | 10,140 | 10,478 |
| Kr-pot-pot-4 | 5,509,254 | 7,093 | 9,987 | 10,322 |
| Kr-pot-pot-5 | 5,538,129 | 7,165 | 10,059 | 10,395 |
| Kr-sp-maz-1 | 6,784,803 | 6,323 | 9,433 |  |
| Kr-sp-maz-2 | 6,056,200 | 6,681 | 9,565 |  |
| Kr-sp-maz-3 | 5,396,006 | 6,090 | 9,216 |  |
| Kr-sp-maz-4 | 7,198,546 | 6,709 | 9,596 |  |
| Kr-pett-berb-1 | 4,781,057 | 3,355 |  |  |
| Kr-pett-berb-2 | 3,850,776 | 3,342 |  |  |
| Kr-xin-1 | 4,908,717 | 3,609 |  | **40of79 matrix** |
| Ae-sp-xin-1 | 5,833,697 | 2,479 |  | snps matrix size: (79, 99922), 15.23% missing sites. |
| Ae-sp-xin-2 | 5,831,358 | 2,690 |  | sequence matrix size: (79, 2107645), 18.51% missing sites. |
| Ae-tet-nov-1 | 2,860,914 | 2,228 |  |  |
| Ae-tet-nov-2 | 4,445,167 | 2,536 |  |  |
| Cich-bim-1 | 3,095,322 | 2,512 |  | **48of63 matrix** |
| Cich-bim-2 | 4,272,750 | 2,627 |  | snps matrix size: (63, 22665), 6.95% missing sites. |
| Kr-gui-sur-1 | 4,926,591 | 3,778 |  | sequence matrix size: (63, 2975799), 5.21% missing sites. |
| Kr-gui-sur-2 | 4,333,213 | 3,787 |  | unlinked snps (63, 7398), 5.90% missing sites |
| Kr-gui1-sinn-1 | 4,011,375 | 3,326 |  |  |
| Kr-gui1-sinn-2 | 5,642,545 | 3,389 |  | **45of59 matrix** |
| Kr-ita-mar-1 | 6,054,820 | 3,532 |  | snps matrix size: (59, 11296), 8.00% missing sites. |
| Kr-ita-mar-4 | 2,033,215 | 3,127 |  | sequence matrix size: (59, 3073420), 4.95% missing sites. |
| Kr-paloe-1 | 4,945,624 | 3,669 |  | unlinked snps (59, 5100), 6.37% missing sites |

Table S3: Observed heterozygosity (H_O_) at each site in the river systems of the Pakaraimas from upstream (low order tributaries) to more downstream sites. See Table S1 for GPS sites of each site. Lowest and highest values within each river system are identified with * and ** respectively.

| Kuribrong upstream | Potaro upstream | Ireng upstream | Mazaruni upstream |
| --- | --- | --- | --- |
| 0.0817* (Kuri-04) | 0.078* (Upot-03) | 0.0516 (UIre-04) | 0.0407* (UMaz-14) |
| 0.1706 (Kuri-03) | 0.0877** (Upot-02) | 0.0432 (UIre-03) | 0.0548** (UMaz-13) |
| 0.2445 (Kuri-02) | 0.0856 (Upot-01) | 0.0642** (UIre-02) | 0.053 (UMaz-12) |
| 0.2661** (Kuri-01) |  | 0.0285* (UIre-01) | 0.056 (UMaz-11) |
|  |  |  | 0.0456 (UMaz-10) |
|  |  |  | 0.0459 (UMaz-09) |
|  |  |  | 0.0511 (UMaz-08) |
|  |  |  | 0.0509 (UMaz-07) |
|  |  |  | 0.0454 (UMaz-06) |
|  |  |  | 0.0533 (UMaz-05) |
|  |  |  | 0.0498 (UMaz-04) |
|  |  |  | 0.0476 (UMaz-03) |
|  |  |  | 0.0421 (UMaz-02) |
|  |  |  | 0.0407* (UMaz-01) |
| Kuribrong downstream | **Potaro downstream** | **Ireng downstream** | **Mazaruni downstream** |

Table S45: Genetic distance, Hudson’s (F_ST_) for *Krobia potaroensis.* between sampling sites in the Pakaraima Mountains of western Guyana (see Table S1) based on 11,173 biallelic SNPs. brown = the Kuribrong River, green= the upper Ireng River, red = the upper Mazaruni River, and yellow = the upper Potaro River. Within-river genetic distances are bordered by a single line, while between-river genetic distances are bordered with a double line.

|  | Kuri-01 | Kuri-02 | Kuri-03 | Kuri-04 | UIre-01 | UIre-02 | | UIre-03 | | UIre-04 | | UMaz-01 | | UMaz-02 | | UMaz-03 | | UMaz-04 | | UMaz-05 | | UMaz-06 | | UMaz-07 | | UMaz-08 | | UMaz-09 | | UMaz-10 | | UMaz-11 | | UMaz-12 | | UMaz-13 | | UMaz-14 | | UPot-01 | | UPot-02 | |
| --- | --- | --- | --- | --- | --- | --- | --- | --- | --- | --- | --- | --- | --- | --- | --- | --- | --- | --- | --- | --- | --- | --- | --- | --- | --- | --- | --- | --- | --- | --- | --- | --- | --- | --- | --- | --- | --- | --- | --- | --- | --- | --- | --- |
| Kuri-01 | x |  |  |  |  |  | |  | |  | |  | |  | |  | |  | |  | |  | |  | |  | |  | |  | |  | |  | |  | |  | |  | |  | |
| Kuri-02 | 0.11 | x |  |  |  |  | |  | |  | |  | |  | |  | |  | |  | |  | |  | |  | |  | |  | |  | |  | |  | |  | |  | |  | |
| Kuri-03 | 0.08 | 0.01 | x |  |  |  | |  | |  | |  | |  | |  | |  | |  | |  | |  | |  | |  | |  | |  | |  | |  | |  | |  | |  | |
| Kuri-04 | 0.17 | 0.12 | 0.05 | x |  |  | |  | |  | |  | |  | |  | |  | |  | |  | |  | |  | |  | |  | |  | |  | |  | |  | |  | |  | |
| UIre-01 | 0.24 | 0.22 | 0.21 | 0.29 | x |  | |  | |  | |  | |  | |  | |  | |  | |  | |  | |  | |  | |  | |  | |  | |  | |  | |  | |  | |
| UIre-02 | 0.20 | 0.17 | 0.12 | 0.19 | 0.17 | | x | |  | |  | |  | |  | |  | |  | |  | |  | |  | |  | |  | |  | |  | |  | |  | |  | |  | |  |
| UIre-03 | 0.22 | 0.19 | 0.14 | 0.24 | 0.20 | 0.02 | | x | |  | |  | |  | |  | |  | |  | |  | |  | |  | |  | |  | |  | |  | |  | |  | |  | |  | |
| UIre-04 | 0.20 | 0.18 | 0.12 | 0.19 | 0.18 | 0.0 | | 0.04 | | x | |  | |  | |  | |  | |  | |  | |  | |  | |  | |  | |  | |  | |  | |  | |  | |  | |
| UMaz-01 | 0.23 | 0.20 | 0.16 | 0.26 | 0.32 | 0.16 | | 0.22 | | 0.19 | | x | |  | |  | |  | |  | |  | |  | |  | |  | |  | |  | |  | |  | |  | |  | |  | |
| UMaz-02 | 0.22 | 0.21 | 0.15 | 0.24 | 0.30 | 0.16 | | 0.22 | | 0.18 | | 0.0 | | x | |  | |  | |  | |  | |  | |  | |  | |  | |  | |  | |  | |  | |  | |  | |
| UMaz-03 | 0.21 | 0.19 | 0.14 | 0.21 | 0.25 | 0.14 | | 0.19 | | 0.15 | | 0.03 | | 0.02 | | x | |  | |  | |  | |  | |  | |  | |  | |  | |  | |  | |  | |  | |  | |
| UMaz-04 | 0.22 | 0.19 | 0.14 | 0.22 | 0.27 | 0.13 | | 0.18 | | 0.15 | | 0.0 | | 0.0 | | 0.0 | | x | |  | |  | |  | |  | |  | |  | |  | |  | |  | |  | |  | |  | |
| UMaz-05 | 0.22 | 0.20 | 0.14 | 0.23 | 0.28 | 0.14 | | 0.20 | | 0.16 | | 0.0 | | 0.01 | | 0.02 | | 0.0 | | x | |  | |  | |  | |  | |  | |  | |  | |  | |  | |  | |  | |
| UMaz-06 | 0.22 | 0.20 | 0.14 | 0.23 | 0.29 | 0.14 | | 0.21 | | 0.16 | | 0.0 | | 0.0 | | 0.0 | | 0.0 | | 0.0 | | x | |  | |  | |  | |  | |  | |  | |  | |  | |  | |  | |
| UMaz-07 | 0.22 | 0.20 | 0.13 | 0.23 | 0.29 | 0.14 | | 0.18 | | 0.17 | | 0.0 | | 0.0 | | 0.0 | | 0.0 | | 0.0 | | 0.0 | | x | |  | |  | |  | |  | |  | |  | |  | |  | |  | |
| UMaz-08 | 0.21 | 0.19 | 0.13 | 0.22 | 0.25 | 0.12 | | 0.17 | | 0.13 | | 0.0 | | 0.0 | | 0.0 | | 0.0 | | 0.0 | | 0.0 | | 0.0 | | x | |  | |  | |  | |  | |  | |  | |  | |  | |
| UMaz-09 | 0.22 | 0.20 | 0.15 | 0.24 | 0.29 | 0.15 | | 0.20 | | 0.17 | | 0.0 | | 0.02 | | 0.02 | | 0.0 | | 0.0 | | 0.0 | | 0.0 | | 0.0 | | x | |  | |  | |  | |  | |  | |  | |  | |
| UMaz-10 | 0.22 | 0.20 | 0.15 | 0.23 | 0.28 | 0.15 | | 0.20 | | 0.17 | | 0.01 | | 0.03 | | 0.02 | | 0.0 | | 0.01 | | 0.0 | | 0.0 | | 0.0 | | 0.0 | | x | |  | |  | |  | |  | |  | |  | |
| UMaz-11 | 0.22 | 0.19 | 0.15 | 0.24 | 0.27 | 0.14 | | 0.20 | | 0.17 | | 0.02 | | 0.03 | | 0.03 | | 0.0 | | 0.0 | | 0.0 | | 0.0 | | 0.0 | | 0.02 | | 0.02 | | x | |  | |  | |  | |  | |  | |
| UMaz-12 | 0.20 | 0.17 | 0.13 | 0.20 | 0.23 | 0.12 | | 0.17 | | 0.14 | | 0.03 | | 0.03 | | 0.02 | | 0.01 | | 0.02 | | 0.02 | | 0.02 | | 0.0 | | 0.03 | | 0.02 | | 0.0 | | x | |  | |  | |  | |  | |
| UMaz-13 | 0.21 | 0.18 | 0.13 | 0.20 | 0.23 | 0.12 | | 0.17 | | 0.13 | | 0.03 | | 0.02 | | 0.02 | | 0.0 | | 0.01 | | 0.01 | | 0.02 | | 0.0 | | 0.02 | | 0.02 | | 0.01 | | 0.0 | | x | |  | |  | |  | |
| UMaz-14 | 0.22 | 0.20 | 0.15 | 0.23 | 0.27 | 0.15 | | 0.20 | | 0.16 | | 0.06 | | 0.03 | | 0.02 | | 0.0 | | 0.03 | | 0.0 | | 0.02 | | 0.0 | | 0.03 | | 0.03 | | 0.04 | | 0.03 | | 0.02 | | x | |  | |  | |
| UPot-01 | 0.17 | 0.14 | 0.07 | 0.12 | 0.18 | 0.02 | | 0.08 | | 0.04 | | 0.10 | | 0.09 | | 0.09 | | 0.07 | | 0.08 | | 0.08 | | 0.07 | | 0.06 | | 0.09 | | 0.09 | | 0.10 | | 0.07 | | 0.05 | | 0.08 | | x | |  | |
| UPot-02 | 0.18 | 0.14 | 0.09 | 0.15 | 0.18 | 0.05 | | 0.12 | | 0.07 | | 0.12 | | 0.11 | | 0.10 | | 0.09 | | 0.10 | | 0.10 | | 0.10 | | 0.08 | | 0.11 | | 0.11 | | 0.08 | | 0.07 | | 0.06 | | 0.11 | | 0.0 | | x | |
| UPot-03 | 0.20 | 0.16 | 0.10 | 0.18 | 0.20 | 0.06 | | 0.13 | | 0.09 | | 0.11 | | 0.11 | | 0.11 | | 0.09 | | 0.09 | | 0.10 | | 0.09 | | 0.08 | | 0.10 | | 0.10 | | 0.08 | | 0.07 | | 0.06 | | 0.11 | | 0.01 | | 0.0 | |


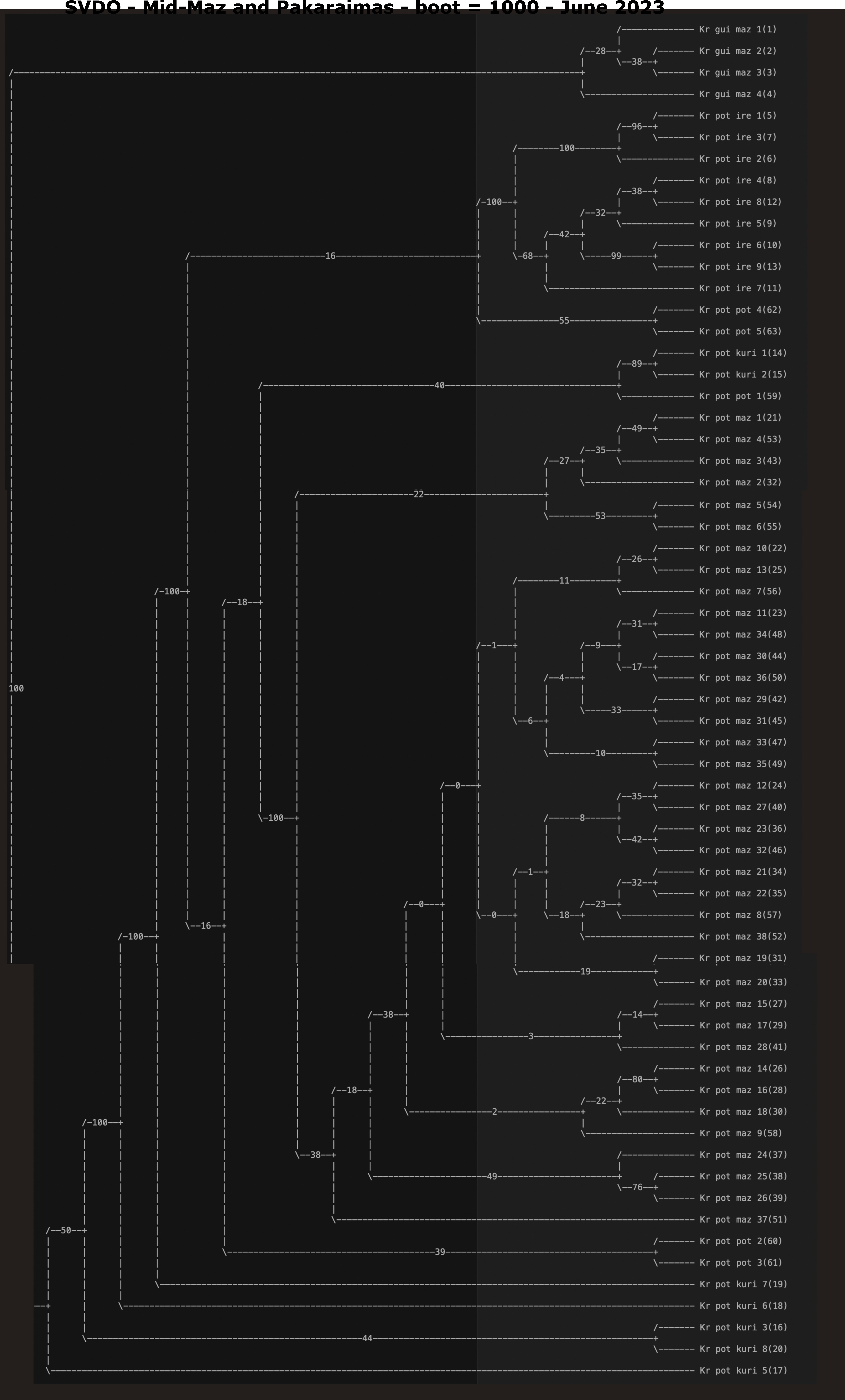


Figure S18: SVDQuartets tree (Swofford 2002; Chifman and Kubatko 2014) for *Krobia potaroensis* individuals in the Pakaraima Mountains of western Guyana. Sample names are as in Table S1. Tree was generated using a matrix of 2,975,799 bp from 10,366 loci. Robustness of relationships at each node was assessed through 1000 bootstrap replicates.

Additional analyses using alternative thresholds of missing data:

Matrix with loci from at least 15 (of 59) individuals (Figs S2 and S4):

6,515 unlinked SNPs with 15.8% missing data.

Matrix with loci from at least 30 (of 59) individuals (Figs S3 and S5)::

5,895 unlinked SNPs with 10.8% missing data.


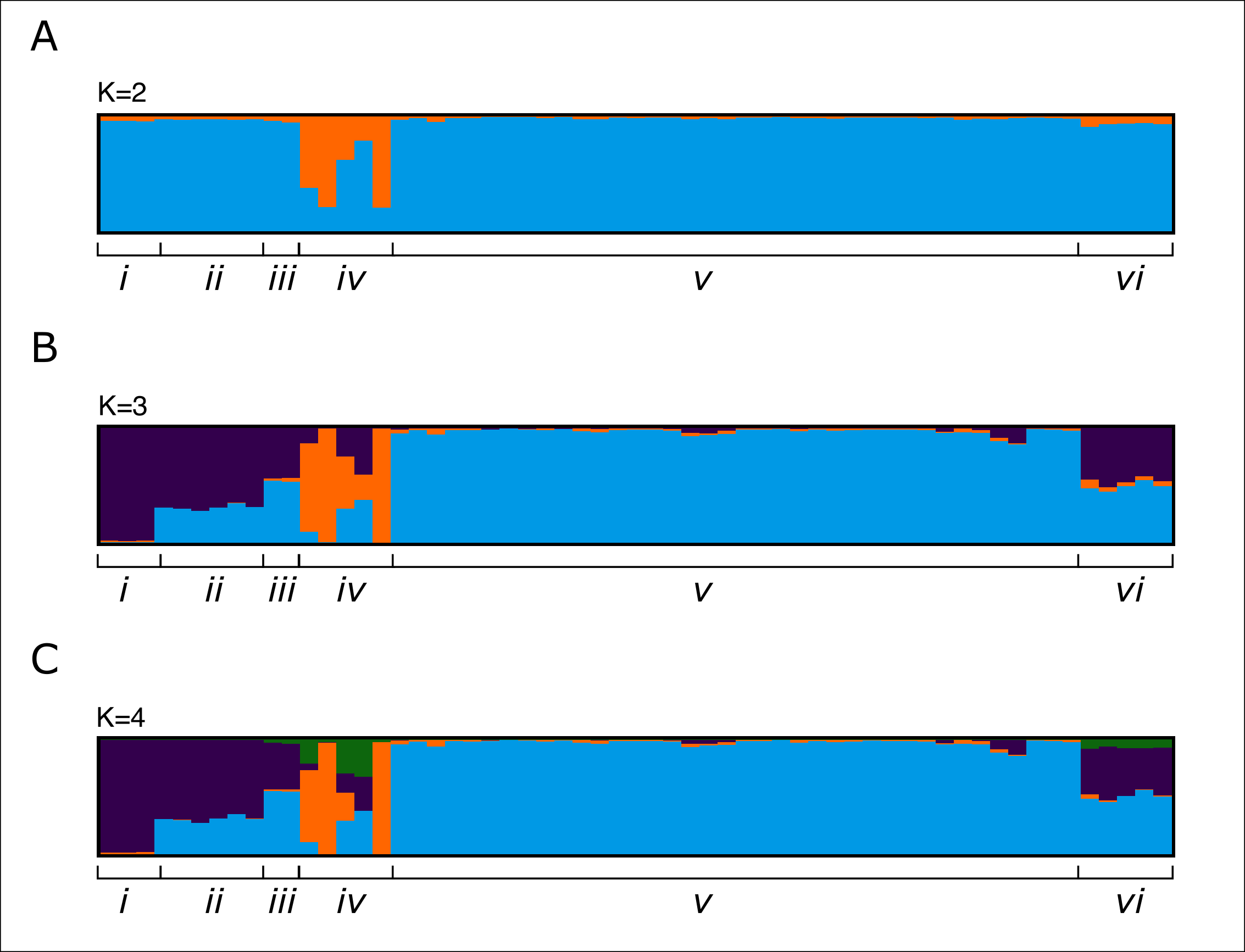


Figure S2: STRUCTURE plots for the 6,516 unlinked SNPs for loci present in at least 15 of 59 *Krobia potaroensis* (n=59) in the Pakaraima Rivers of western Guyana. Following a burn-in of 100,000 MCMC iterations each STRUCTURE (Pritchard et al 2000) run was conducted with 1,000,000 MCMC iterations with 10 independent searches of each K value ranging from 1 to 7; parallelized using Strauto (v1.0 Chhatre and Emerson 2017). Convergence was assessed with Structure Harvester (Earl and Von Holdt 2012) and results were summarized using the Clumpak pipeline (Jakobsson and Rosenberg 2007; Kopelman et al. 2015). Cluster assignments are shown for values of A: K=2, B: K=3, and C: K=4. Sample order: i) lower Ireng River (N=3), ii) upper Ireng River (N=5), iii+vi) upper Potaro River (N=2 and 5 respectively), and v) the upper Mazaruni River (n=38).


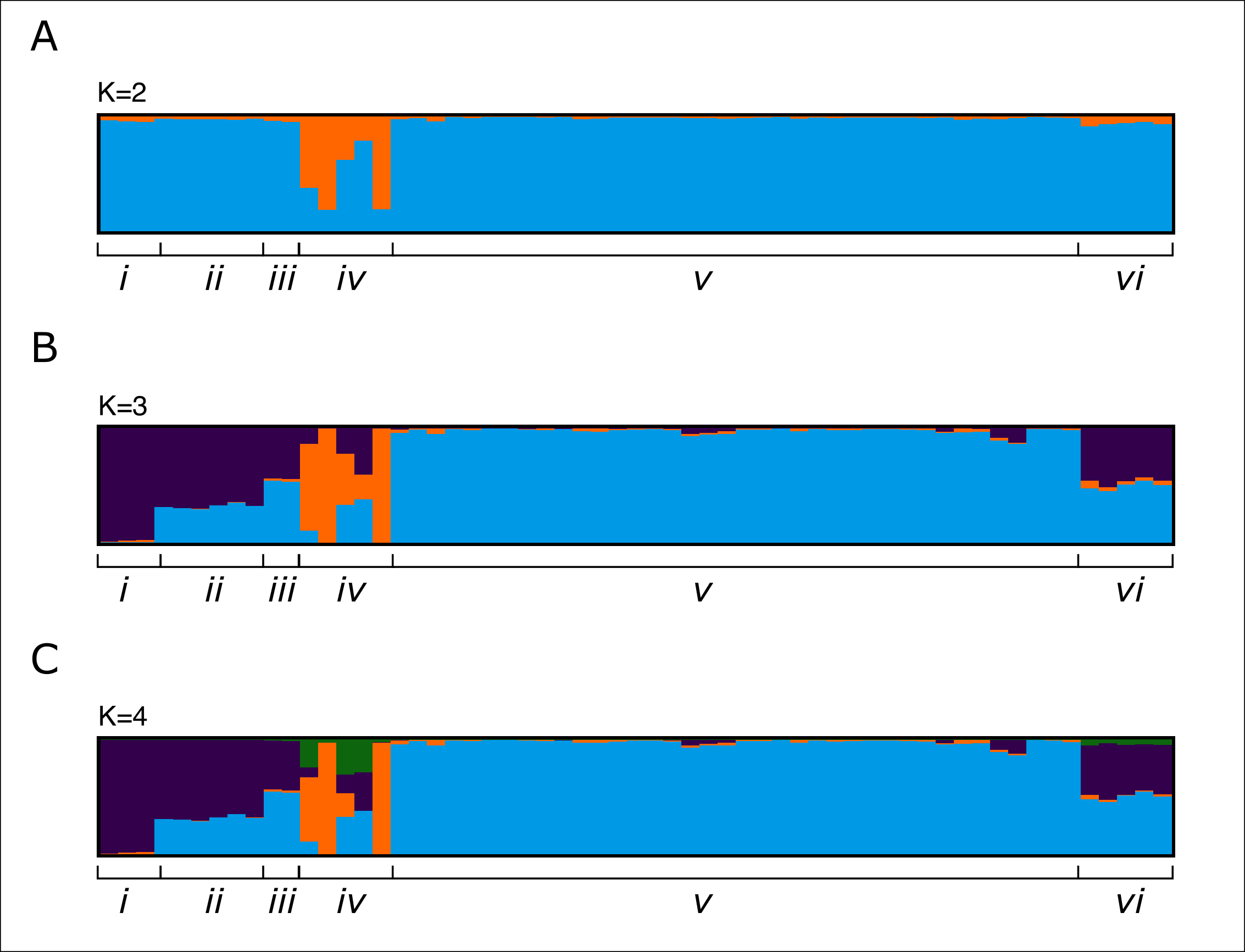


Figure S3: STRUCTURE plots for the 5,895 unlinked SNPs for loci present in at least 30 of 59 *Krobia potaroensis* (n=59) in the Pakaraima Rivers of western Guyana. Following a burn-in of 100,000 MCMC iterations each STRUCTURE (Pritchard et al 2000) run was conducted with 1,000,000 MCMC iterations with 10 independent searches of each K value ranging from 1 to 7; parallelized using Strauto (v1.0 Chhatre and Emerson 2017). Convergence was assessed with Structure Harvester (Earl and Von Holdt 2012) and results were summarized using the Clumpak pipeline (Jakobsson and Rosenberg 2007; Kopelman et al. 2015). Cluster assignments are shown for values of A: K=2, B: K=3, and C: K=4. Sample order: i) lower Ireng River (N=3), ii) upper Ireng River (N=5), iii+vi) upper Potaro River (N=2 and 5 respectively), and v) the upper Mazaruni River (n=38).

Figure S4: Genetic principal components analysis for *Krobia potaroensis* (n=59) from the upland rivers of the Pakaraimas Mountains in western Guyana. Analysis conducted in adegenet v2.1.10 (Jombart and Ahmed, 2011) for 6,515 unlinked SNPs with 15.8% missing data. River systems are abbreviated: UIre = Upper Ireng River, UPot = Upper Potaro River, Kuri = Kuribrong River, and UMaz = Upper Mazaruni River.

Figure S5: Genetic principal components analysis for *Krobia potaroensis* (n=59) from the upland rivers of the Pakaraimas Mountains in western Guyana. Analysis conducted in adegenet v2.1.10 (Jombart and Ahmed, 2011) for 5,895 unlinked SNPs with 10.8% missing data. River systems are abbreviated: UIre = Upper Ireng River, UPot = Upper Potaro River, Kuri = Kuribrong River, and UMaz = Upper Mazaruni River.

Table S5: Model comparison in *dadi-cli* (Gutenkunst et al. 2009; Huang et al. 2021) for six demographic models (Portik et al. 2017) describing the pairwise genetic relationships between five populations of *Krobia potaroensis* in the Pakaraimas of western Guyana. The preferred model (lowest AIC) is listed first for each pair and shown in bold. ΔAIC is relative to the best-supported model (reference = 0.00). Support ratings: *** = ΔAIC > 10 for all alternatives (strong support); * = marginal support (ΔAIC < 10 for ≥1 alternative). † Ireng–Middle-Maz: sec_contact_asym_mig (ΔAIC = 2.26) and no_mig_size (ΔAIC = 3.63) are close competitors; interpret IM parameters with caution.

| **Population pair** | **Model** | **log-likelihood** | **AIC** | **ΔAIC** | **Support** |
| --- | --- | --- | --- | --- | --- |
| **Ireng–Potaro** | **IM** | **-202.33** | **416.66** | **0.00 (ref)** | ******* |
|  | no_mig | -240.43 | 486.85 | 70.19 |  |
|  | no_mig_size | -214.71 | 441.41 | 24.76 |  |
|  | sec_contact_asym_mig | -210.39 | 432.77 | 16.12 |  |
|  | sec_contact_sym_mig | -213.29 | 436.57 | 19.91 |  |
|  | split_mig | -217.74 | 443.48 | 26.82 |  |
| **Ireng–Kuribrong** | **IM** | **-162.19** | **336.37** | **0.00 (ref)** | ******* |
|  | no_mig | -297.78 | 601.57 | 265.20 |  |
|  | no_mig_size | -278.65 | 569.30 | 232.93 |  |
|  | sec_contact_asym_mig | -172.08 | 356.16 | 19.79 |  |
|  | sec_contact_sym_mig | -189.31 | 388.63 | 52.26 |  |
|  | split_mig | -189.27 | 386.55 | 50.18 |  |
| **Ireng–Upper-Maz** | **IM** | **-557.70** | **1127.41** | **0.00 (ref)** | ******* |
|  | no_mig | -787.98 | 1581.96 | 454.55 |  |
|  | no_mig_size | -575.50 | 1162.98 | 35.59 |  |
|  | sec_contact_asym_mig | -719.08 | 1450.15 | 322.75 |  |
|  | sec_contact_sym_mig | -748.86 | 1507.72 | 380.31 |  |
|  | split_mig | -753.55 | 1515.10 | 387.69 |  |
| **Ireng–Middle-Maz** | **IM** | **-154.16** | **320.31** | **0.00 (ref)** | ***†** |
|  | no_mig | -256.58 | 519.16 | 198.85 |  |
|  | no_mig_size | -155.97 | 323.94 | 3.63 |  |
|  | sec_contact_asym_mig | -155.28 | 322.57 | 2.26 |  |
|  | sec_contact_sym_mig | -167.29 | 344.58 | 24.27 |  |
|  | split_mig | -179.04 | 366.09 | 45.78 |  |
| **Potaro–Kuribrong** | **IM** | **-151.42** | **314.84** | **0.00 (ref)** | ******* |
|  | no_mig | -299.32 | 604.64 | 289.80 |  |
|  | no_mig_size | -283.64 | 579.28 | 264.44 |  |
|  | sec_contact_asym_mig | -154.66 | 321.32 | 6.48 |  |
|  | sec_contact_sym_mig | -170.02 | 350.04 | 35.19 |  |
|  | split_mig | -170.01 | 348.02 | 33.18 |  |
| **Potaro–Upper-Maz** | **IM** | **-481.40** | **974.80** | **0.00 (ref)** | ******* |
|  | no_mig | -741.80 | 1489.61 | 514.80 |  |
|  | no_mig_size | -510.82 | 1033.63 | 58.83 |  |
|  | sec_contact_asym_mig | -667.06 | 1346.12 | 371.31 |  |
|  | sec_contact_sym_mig | -715.05 | 1440.09 | 465.29 |  |
|  | split_mig | -727.90 | 1463.79 | 488.99 |  |
| **Potaro–Middle-Maz** | **IM** | **-139.85** | **291.70** | **0.00 (ref)** | ******* |
|  | no_mig | -243.87 | 493.74 | 202.04 |  |
|  | no_mig_size | -149.90 | 311.80 | 20.10 |  |
|  | sec_contact_asym_mig | -151.78 | 315.56 | 23.85 |  |
|  | sec_contact_sym_mig | -178.57 | 367.14 | 75.44 |  |
|  | split_mig | -190.67 | 389.34 | 97.63 |  |
| **Kuribrong–Upper-Maz** | **IM** | **-454.06** | **920.13** | **0.00 (ref)** | ******* |
|  | no_mig | -850.97 | 1707.95 | 787.82 |  |
|  | no_mig_size | -520.07 | 1052.14 | 132.01 |  |
|  | sec_contact_asym_mig | -628.42 | 1268.84 | 348.72 |  |
|  | sec_contact_sym_mig | -783.11 | 1576.23 | 656.10 |  |
|  | split_mig | -783.11 | 1574.22 | 654.10 |  |
| **Kuribrong–Middle-Maz** | **IM** | **-114.29** | **240.58** | **0.00 (ref)** | ******* |
|  | no_mig | -229.40 | 464.80 | 224.21 |  |
|  | no_mig_size | -196.81 | 405.61 | 165.03 |  |
|  | sec_contact_asym_mig | -124.75 | 261.49 | 20.91 |  |
|  | sec_contact_sym_mig | -160.98 | 331.97 | 91.38 |  |
|  | split_mig | -162.97 | 333.95 | 93.36 |  |
| **Upper-Maz–Middle-Maz** | **no_mig_size** | **-464.42** | **940.83** | **0.00 (ref)** | ******* |
|  | IM | -499.90 | 1011.80 | 70.97 |  |
|  | no_mig | -615.26 | 1236.52 | 295.69 |  |
|  | sec_contact_asym_mig | -513.18 | 1038.36 | 97.53 |  |
|  | sec_contact_sym_mig | -514.02 | 1038.05 | 97.22 |  |
|  | split_mig | -514.85 | 1037.71 | 96.87 |  |

Table S6: Demographic parameters for preferred models. Best fit parameter estimates, and 95% confidence intervals obtained via parametric bootstrapping in dadi-cli. Five populations of *Krobia potaroensis* from the Pakaraimas Rivers (upper Mazaruni, upper Potaro River, Upper Ireng River, and Kuribrong River) and the middle Mazaruni River (n=63). For IM (isolation-with-migration) models: s = ancestral population size fraction retained by Ireng at split; nu1, nu2 = relative sizes of daughter populations; T = divergence time (years, assuming generation time and mutation rate as described in Methods); m12, m21 = migration rates (proportion of population replaced per generation). For no_mig_size (Upper-Maz–Middle-Maz): nu1a, nu2a = initial sizes post-split; nu1b, nu2b = final sizes; T1, T2 = durations of first and second epochs (years).

| **Pair** | **Model** | **Parameter** | **Na** | **BestFit_bio** | **Lower_bio** | **Upper_bio** |
| --- | --- | --- | --- | --- | --- | --- |
| Ireng-Potaro | IM | s | 7,555 | 0.01 | 0.01 | 0.02 |
| Ireng-Potaro |  | nu1 | 7,555 | 75,552 | 3522.24 | 1620602.33 |
| Ireng-Potaro |  | nu2 | 7,555 | 1,378 | 654.21 | 2903.83 |
| Ireng-Potaro |  | T | 7,555 | 804 | 596.38 | 1085.16 |
| Ireng-Potaro |  | m12 | 7,555 | 7.72 x10^-5^ | 3.13 x10^-5^ | 1.90 x10^-4^ |
| Ireng-Potaro |  | m21 | 7,555 | 6.06 x10^-4^ | 3.34 x10^-4^ | 1.90 x10^-4^ |
| Ireng-Kuribrong | IM | s | 22,884 | 0.01 | 0.01 | 0.01 |
| Ireng-Kuribrong |  | nu1 | 22,884 | 4,528 | 2,895 | 7,084 |
| Ireng-Kuribrong |  | nu2 | 22,884 | 227,696 | 15,787 | 3,283,979 |
| Ireng-Kuribrong |  | T | 22,884 | 2,697 | 2,646 | 2,749 |
| Ireng-Kuribrong |  | m12 | 22,884 | 5.31 x10^-6^ | 1.34 x10^-6^ | 2.10 x10^-5^ |
| Ireng-Kuribrong |  | m21 | 22,884 | 1.93 x10^-4^ | 1.81 x10^-4^ | 2.04 x10^-4^ |
| Ireng-Upper-Maz | IM | s | 6,603 | 0.97 | 0.97 | 0.97 |
| Ireng-Upper-Maz |  | nu1 | 6,603 | 630 | 498 | 796 |
| Ireng-Upper-Maz |  | nu2 | 6,603 | 66,026 | 36,040 | 120,963 |
| Ireng-Upper-Maz |  | T | 6,603 | 1,421 | 1,421 | 1,421 |
| Ireng-Upper-Maz |  | m12 | 6,603 | 1.39 x10^-4^ | 1.39 x10^-4^ | 1.39 x10^-4^ |
| Ireng-Upper-Maz |  | m21 | 6,603 | 3.52 x10^-5^ | 3.52 x10^-5^ | 5.47 x10^-5^ |
| Ireng-Middle-Maz | IM | s | 40,791 | 0.16 | 0.16 | 0.17 |
| Ireng-Middle-Maz |  | nu1 | 40,791 | 2,069 | 2,062 | 2,075 |
| Ireng-Middle-Maz |  | nu2 | 40,791 | 35,999 | 32,231 | 40,208 |
| Ireng-Middle-Maz |  | T | 40,791 | 71,321 | 71,246 | 71,396 |
| Ireng-Middle-Maz |  | m12 | 40,791 | 3.62 x10^-6^ | 3.14 x10^-6^ | 4.17 x10^-6^ |
| Ireng-Middle-Maz |  | m21 | 40,791 | 4.31 x10^-7^ | 1.75 x1010^-7^ | 1.06 x1010^-6^ |
| Potaro-Kuribrong | IM | s | 23,050 | 0.03 | 0.01 | 0.07 |
| Potaro-Kuribrong |  | nu1 | 23,050 | 3,131 | 537 | 18,254 |
| Potaro-Kuribrong |  | nu2 | 23,050 | 227,496 | 17 | 3,108,747,374 |
| Potaro-Kuribrong |  | T | 23,050 | 3,739 | 3,409 | 4,099 |
| Potaro-Kuribrong |  | m12 | 23,050 | 1.53 x10^-5^ | 1.72 x10^-6^ | 1.37 x10^-4^ |
| Potaro-Kuribrong |  | m21 | 23,050 | 2.17 x10^-4^ | 2.12 x10^-4^ | 2.22 x10^-4^ |
| Potaro-Upper-Maz | IM | s | 8,215 | 0.99 | 0.99 | 0.99 |
| Potaro-Upper-Maz |  | nu1 | 8,215 | 1,251 | 1,000 | 1,566 |
| Potaro-Upper-Maz |  | nu2 | 8,215 | 82,154 | 21552 | 313,166 |
| Potaro-Upper-Maz |  | T | 8,215 | 1,292 | 1,292 | 1,293 |
| Potaro-Upper-Maz |  | m12 | 8,215 | 6.02 x10^-4^ | 6.00 x10^-4^ | 6.03 x10^-4^ |
| Potaro-Upper-Maz |  | m21 | 8,215 | 3.40 x10^-5^ | 1.79 x10^-5^ | 6.46 x10^-5^ |
| Potaro-Middle-Maz | IM | s | 53,399 | 0.8 | 0.79 | 0.8 |
| Potaro-Middle-Maz |  | nu1 | 53,399 | 1,736 | 1,732 | 1,739 |
| Potaro-Middle-Maz |  | nu2 | 53,399 | 57,337 | 48,978 | 67,122 |
| Potaro-Middle-Maz |  | T | 53,399 | 38,183 | 38,115 | 38,250 |
| Potaro-Middle-Maz |  | m12 | 53,399 | 3.49 x10^-6^ | 2.85 x10^-6^ | 4.26 x10^-6^ |
| Potaro-Middle-Maz |  | m21 | 53,399 | 7.29 x10^-8^ | 4.68 x10^-11^ | 1.05 x10^-4^ |
| Kuribrong-Upper-Maz | IM | s | 20,083 | 0.99 | 0.99 | 0.99 |
| Kuribrong-Upper-Maz |  | nu1 | 20,083 | 200,826 | 403 | 99,964,962 |
| Kuribrong-Upper-Maz |  | nu2 | 20,083 | 17,791 | 13,772 | 22,981 |
| Kuribrong-Upper-Maz |  | T | 20,083 | 3,244 | 3,158 | 3,332 |
| Kuribrong-Upper-Maz |  | m12 | 20,083 | 1.29 x10^-4^ | 1.28 x10^-4^ | 1.31 x10^-4^ |
| Kuribrong-Upper-Maz |  | m21 | 20,083 | 1.87 x10^-6^ | 9.66 x10^-7^ | 3.62 x10^-6^ |
| Kuribrong-Middle-Maz | IM | s | 38,057 | 0.04 | 0.04 | 0.04 |
| Kuribrong-Middle-Maz |  | nu1 | 38,057 | 16,639 | 15,440 | 17,932 |
| Kuribrong-Middle-Maz |  | nu2 | 38,057 | 35,124 | 31,748 | 38,859 |
| Kuribrong-Middle-Maz |  | T | 38,057 | 83,903 | 83,863 | 83,942 |
| Kuribrong-Middle-Maz |  | m12 | 38,057 | 5.19 x10^-6^ | 4.75 x10^-6^ | 5.68 x10^-6^ |
| Kuribrong-Middle-Maz |  | m21 | 38,057 | 6.58 x10^-7^ | 3.72 x10^-7^ | 1.17 x10^-6^ |
| Upper-Maz-Middle-Maz | no_mig_size | nu1a | 62,176 | 2,097 | 1,412 | 3,117 |
| Upper-Maz-Middle-Maz |  | nu2a | 62,176 | 11,084 | 7,462 | 16,463 |
| Upper-Maz-Middle-Maz |  | T1 | 62,176 | 1,243,527 | 24,781 | 62,400,321 |
| Upper-Maz-Middle-Maz |  | nu1b | 62,176 | 332370.93 | 190815.75 | 578937.71 |
| Upper-Maz-Middle-Maz |  | nu2b | 62,176 | 6,988 | 4,566 | 10,697 |
| Upper-Maz-Middle-Maz |  | T2 | 62,176 | 461 | 389 | 545 |

Literature cited:

Chhatre, V. E., & Emerson, K. J. (2017). StrAuto: Automation and parallelization of STRUCTURE analysis. BMC Bioinformatics, 18(1), 192. https://doi.org/10.1186/s12859-017-1593-0

Chifman, J., & Kubatko, L. (2014). Quartet Inference from SNP Data Under the Coalescent Model. Bioinformatics, 30(23), 3317–3324. https://doi.org/10.1093/bioinformatics/btu530

Earl, D. A., & vonHoldt, B. M. (2012). STRUCTURE HARVESTER: A website and program for visualizing STRUCTURE output and implementing the Evanno method. Conservation Genetics Resources, 4(2), 359–361. https://doi.org/10.1007/s12686-011-9548-7

Eaton, D. A. R., & Overcast, I. (2020). ipyrad: Interactive assembly and analysis of RADseq datasets. Bioinformatics, 36(8), 2592–2594. https://doi.org/10.1093/bioinformatics/btz966

Gutenkunst, R. N., Hernandez, R. D., Williamson, S. H., & Bustamante, C. D. (2009). Inferring the Joint Demographic History of Multiple Populations from Multidimensional SNP Frequency Data. PLoS Genetics, 5(10), e1000695. https://doi.org/10.1371/journal.pgen.1000695 Jakobsson, M., & Rosenberg, N. A. (2007). CLUMPP: A cluster matching and permutation program for dealing with label switching and multimodality in analysis of population structure. Bioinformatics, 23(14), 1801–1806. https://doi.org/10.1093/bioinformatics/btm233

Huang, X., Fortier, A. L., Coffman, A. J., Struck, T. J., Irby, M. N., James, J. E., León-Burguete, J. E., Ragsdale, A. P., & Gutenkunst, R. N. (2021). Inferring Genome-Wide Correlations of Mutation Fitness Effects between Populations. Molecular Biology and Evolution, 38(10), 4588–4602. https://doi.org/10.1093/molbev/msab162

Jombart, T., & Ahmed, I. (2011). adegenet 1.3-1: New tools for the analysis of genome-wide SNP data. Bioinformatics, 27(21), 3070–3071. https://doi.org/10.1093/bioinformatics/btr521

Kopelman, N. M., Mayzel, J., Jakobsson, M., Rosenberg, N. A., & Mayrose, I. (2015). Clumpak: A program for identifying clustering modes and packaging population structure inferences across K. Molecular Ecology Resources, 15(5), 1179–1191. https://doi.org/10.1111/1755-0998.12387

Portik, D. M., Leaché, A. D., Rivera, D., Barej, M. F., Burger, M., Hirschfeld, M., Rödel, M., Blackburn, D. C., & Fujita, M. K. (2017). Evaluating mechanisms of diversification in a Guineo‐Congolian tropical forest frog using demographic model selection. Molecular Ecology, 26(19), 5245–5263. https://doi.org/10.1111/mec.14266

Pritchard, J. K., Stephens, M., & Donnelly, P. (2000). Inference of Population Structure Using Multilocus Genotype Data. Genetics, 155(2), 945–959.

Swofford, D. (2002). PAUP*. Phylogenetic analysis using parsimony (and other methods), version 4.0. Sinauer Associates, Sunderland, Massachusetts.
